# Gait analysis comparison between manual marking, 2D pose estimation algorithms, and 3D marker-based system

**DOI:** 10.3389/fresc.2023.1238134

**Published:** 2023-09-06

**Authors:** Dimitrios Menychtas, Nikolaos Petrou, Ioannis Kansizoglou, Erasmia Giannakou, Athanasios Grekidis, Antonios Gasteratos, Vassilios Gourgoulis, Eleni Douda, Ilias Smilios, Maria Michalopoulou, Georgios Ch. Sirakoulis, Nikolaos Aggelousis

**Affiliations:** ^^1^^ Biomechanics Laboratory, Department of Physical Education and Sports Science, Democritus University of Thrace, Komotini, Greece; ^^2^^ Laboratory of Robotics and Automation, Department of Production and Management Engineering, Democritus University of Thrace, Xanthi, Greece; ^^3^^ Department of Electrical and Computer Engineering, Democritus University of Thrace, Xanthi, Greece

**Keywords:** 2D pose estimation, motion analysis, biomechanics, joint angle comparison, biomechanics video annotation

## Abstract

**Introduction:**

Recent advances in Artificial Intelligence (AI) and Computer Vision (CV) have led to automated pose estimation algorithms using simple 2D videos. This has created the potential to perform kinematic measurements without the need for specialized, and often expensive, equipment. Even though there's a growing body of literature on the development and validation of such algorithms for practical use, they haven't been adopted by health professionals. As a result, manual video annotation tools remain pretty common. Part of the reason is that the pose estimation modules can be erratic, producing errors that are difficult to rectify. Because of that, health professionals prefer the use of tried and true methods despite the time and cost savings pose estimation can offer.

**Methods:**

In this work, the gait cycle of a sample of the elderly population on a split-belt treadmill is examined. The Openpose (OP) and Mediapipe (MP) AI pose estimation algorithms are compared to joint kinematics from a marker-based 3D motion capture system (Vicon), as well as from a video annotation tool designed for biomechanics (Kinovea). Bland-Altman (B-A) graphs and Statistical Parametric Mapping (SPM) are used to identify regions of statistically significant difference.

**Results:**

Results showed that pose estimation can achieve motion tracking comparable to marker-based systems but struggle to identify joints that exhibit small, but crucial motion.

**Discussion:**

Joints such as the ankle, can suffer from misidentification of their anatomical landmarks. Manual tools don't have that problem, but the user will introduce a static offset across the measurements. It is proposed that an AI-powered video annotation tool that allows the user to correct errors would bring the benefits of pose estimation to professionals at a low cost.

## 1. Introduction

The measurement of three-dimensional human kinematics, such as joint angles, position of the limbs, velocity of the motion etc., can identify motion abnormalities early on, as well as guide health professionals during rehabilitation. However, the necessary equipment is not always accessible to the experts or cost-efficient. Time investment, dedicated space, and the requirement for trained operators, force health professionals to be reluctant to go through that process. As a result, a lot of decisions in low-priority cases are not driven by data but rather, they rely on the personal experience and judgment of the expert.

Multiple tools have been developed for kinematic measurements which vary in complexity and cost, with the more accurate ones not necessarily being practical in all situations. For example, the golden standard for motion tracking is biplanar videoradiography for tracking the movement of the bones using X-rays (1). These systems use two X-ray cameras to record the movement of the bones and they can achieve sub-millimeter and sub-degree error (2). However, they can only capture a single joint because of their limited field of view, there is also a high cost associated with them, and the exposure of the person to radiation. As a result, it is impractical for most use cases (1). The de facto standard practice is the use of marker-based optical multicamera systems. They use retroreflective markers that are placed on anatomical landmarks by a trained professional and the person performs tasks inside an area that is visible from all the infra-red cameras. The 2D images that are recorded are triangulated to give the 3D motions of the body (3,4). Though they have higher error than biplanar videoradiography, and they are sensitive to marker placement, they have been widely adopted in both academic and clinical settings, because of their relative affordability and adaptability to most situations.

Both of the methods that were just described require preparation by an expert before each measurement and time-consuming post-processing that needs different expertise, not to mention dedicated indoor space. As such, they are not accessible to most health professionals and sports trainers. More importantly, they are not practical methods for non-critical assessment even if the need is not trivial. To meet this demand, in the last decade, markerless single-camera systems have been tested as alternatives. The camera that popularized this approach was the Kinect (Microsoft Corporation, Redmond, WA, USA). It has an RGB camera and an infrared depth sensor to detect human motions. Kinect was originally released for Microsoft’s 7th generation video game console (Xbox360) to enable interactions without a gamepad. However, it soon found its place as an affordable biomechanics motion tracker in multiple projects that range from ergonomic assessment to biomimetic robotics (5–12)). Nowadays, more devices are available that combine simple 2D cameras with depth sensors and they are used in different projects, such as collaborative robots for industrial environments (13). With the advancement of Artificial Intelligence (AI) and Computer Vision (CV), there’s a new trend of using a standard 2D RGB camera to extract kinematic information. The benefit of using standard video for biomechanics is that data can be recorded easily in any environment with accessible equipment without any particular preparation. Obviously, the main workload falls on the pose estimation algorithm to extract accurate measurements.

On that front, there have been significant advances, and while there’s still a lot of work to be done, the existing software is mature enough for practical applications. The most popular pose estimation algorithm is OpenPose (OP) (14) which uses a Convolutional Neural Network (CNN) to detect keypoints and then constructs a kinematic skeleton of the human body (bottom-up approach). OP is well documented and it has been validated for gait analyses in biomechanics (15) but its biggest limitation is the relatively high computational demands and that it requires coding skills to produce usable metrics. Another pose estimation algorithm that has gained traction is MediaPipe (MP) (16). Strictly speaking, MP is a framework that can incorporate different CV and machine learning algorithms for fast prototyping. In this work, the focus is on the pose estimation algorithm that it uses. MP produces joint angles using fewer resources than OP, however, it uses a less accurate process where the body is identified and then the joints are estimated (top-down approach). Regardless of the specifics, pose estimation algorithms suffer from errors in motions perpendicular to the video’s plane, as well as the fact that the dataset they were trained with, may not have been prepared by experts. Therefore, the joint centers’ locations may be inaccurate (1). It is also worth noting that in their published form, they do not calculate any kinematics, but the locations of the keypoints. As a result, additional software scripts are required by users depending on the application. Though not necessarily a limitation for academic research, this creates an extra barrier for the adoption of this technology by health professionals.

The simplest method to perform gait analyses is with video annotation tools. The process is to record a video and then manually mark the joint centers and calculate the angles based on the number of pixels. A modern open-source software that does that is Kinovea (KV) (www.kinovea.org). A clinician or a sports trainer doesn’t require any additional effort to perform measurements using KV, but there is a significant time investment for each case. It is decently accurate when compared with established motion capture systems (17) though, its errors should always be kept in perspective for clinical applications (18). Despite KV’s limitations, its low cost, portability, and straightforward use, make it an appealing tool for professionals even if more complex systems are available.

In this work, gait kinematics from a marker-based 3D motion capture system, OP, MP, and KV were compared to evaluate accuracy as well as speed of results, ease of use, and cost. In a similar work, Haberkamp et al. (19) compared Kinovea, Openpose, and marker-based motion capture for single-leg squatting in adolescents and, in agreement with the literature, found good agreement in the sagittal plane, but not on the frontal. The advantage of the squatting task was that it required the joints of the lower limb to reach their maximum range and return to the neutral position, allowing for angles to be large enough for identification. However, clinically important assessments may not exhibit large joint angles, but rather a pattern of subtle motions. It is also important to note that during gait, the extremities overlap on the sagittal plane making identification of the joints challenging. This is why the focus here was on a full gait cycle, because it is a more complex motion with smaller but critical joint movements that is more likely to be used as an assessment task in a larger variety of individuals (from adolescents to older people). As such, it is important to evaluate pose estimation tools with a clinically relevant task that can be challenging to measure accurately. The purpose was to identify the system that can be reliably integrated into everyday practice for non-critical cases.

## 2. Methods

### 2.1. Data collection

Seventeen healthy people with a mean age of 69±5 years participated in motion capture sessions. They did not have any mobility limitations or cognitive maladies. All of them reported to be independent in their daily lives and they were generally active. A split-belt treadmill (Bertec Corporation, Columbus, OH, USA) was used and their motions were recorded at 100 Hz with a Vicon (VC) marker-based motion capture system (Vicon Motion Systems Ltd, Oxford, UK) that was using ten infra-red cameras. The motion capture system also had two standard RGB video cameras integrated and synchronized with the rest of the equipment that were recording at 100 Hz as well. The RGB camera that captured motions in the sagittal plane was used for this work.

The study received ethical approval from the Research Ethics Committee of the Democritus University of Thrace (DUTH/EHDE/28061/165) and was in accordance with international ethical rules.

### 2.2. Experimental protocol

**Figure 1:**
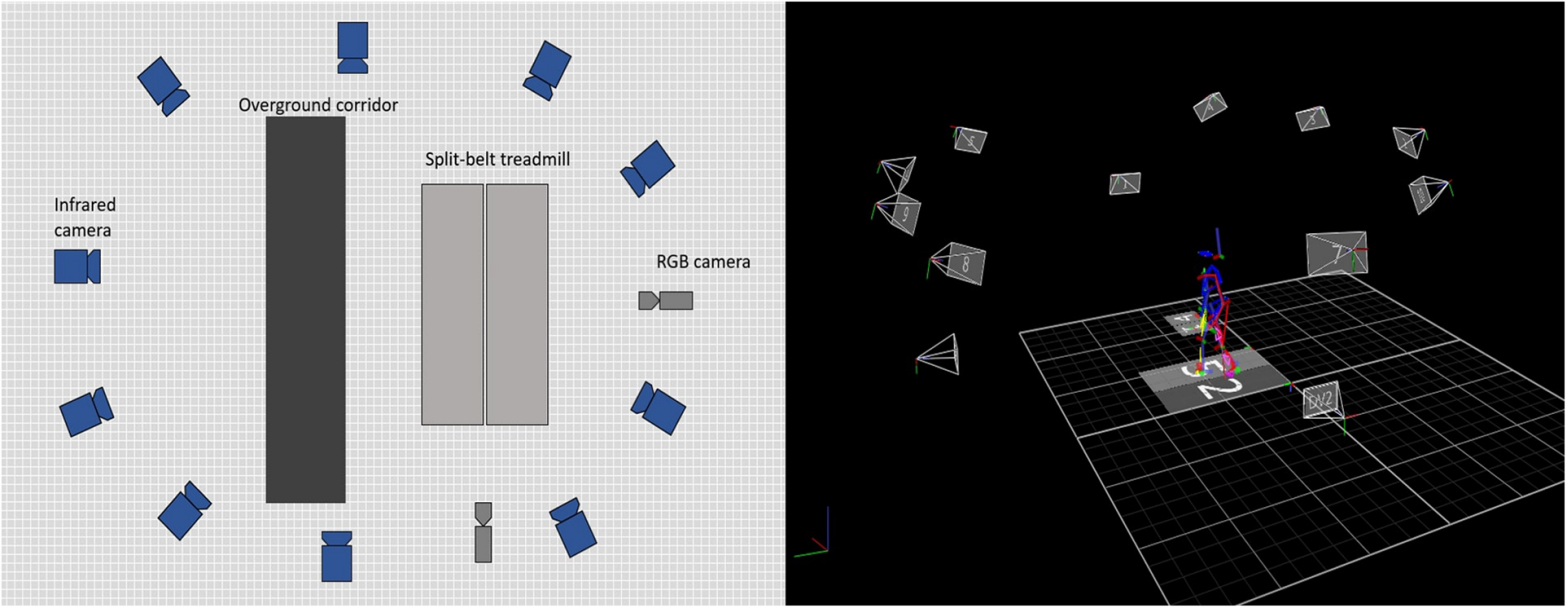
Setup of the equipment. Ten infrared cameras and two RGB cameras were used to record each participant on the overground corridor to calculate their natural gait speed and then, they performed one minute walking on the split-belt treadmill.

The marker set of the Conventional Gait Model version 2.4 (CGM 2.4) was used. It has 57 reflective markers placed on the whole body and it is fully integrated with the system’s software (Nexus 2.14). The participants walked ten times on an overground corridor for ten meters in order to measure their natural gait speed. Figure 1 shows how the lab was set up. Once this was done, they would start walking on the treadmill for a few minutes to familiarize themselves and reach their natural walking speed. Once they were walking comfortably, a full minute of normal gait on the treadmill was recorded for each individual. For this work, a complete gait cycle of the left leg for each person was used. The cycle was defined between two left heel strikes. A heel strike was defined as the moment the heel touched the ground and the treadmill detected a force. In total, 17 gait cycles, one for each person, were used.

### 2.3. Joint angles calculation

The joint angles from the markers of the full body were calculated using the CGM 2.4 algorithm that is integrated into the system’s associated software. From the synchronized RGB video, a gait cycle was selected and the angles of the hip, knee, and ankle were extracted. For OP and MP, custom scripts in Python (ver. 3.9.13) and Matlab (ver. R2020b) (Mathworks Inc., Natick, MA, USA) were written to extract the keypoints, organize the data, and calculate the joint angles in a compatible with the CGM output. The low pass filter that is integrated in Matlab (command: y=lowpass(x,\,fpass, fs)) was employed with a cut-off frequency of 5 Hz to the raw signal.

### 2.4. Manual video annotation with Kinovea

**Figure 2:**
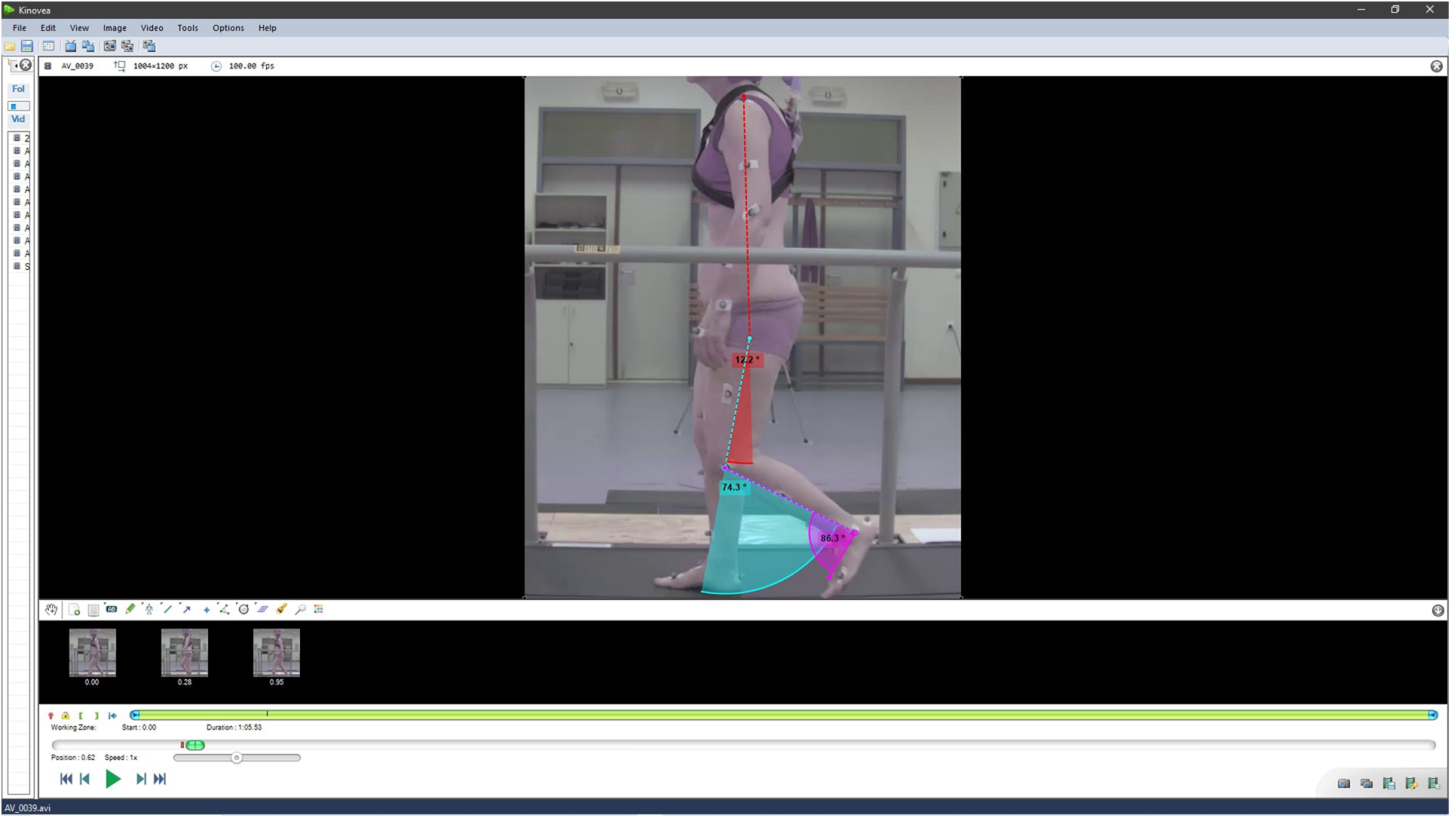
Manual annotation using KV.

The videos were annotated manually using Kinovea (KV) by NP. The angles of the hip, knee, and ankle were exported and compared with the other systems. A gait cycle was selected for each participant to be annotated. Figure 2 shows an example of a manually annotated frame. Assuming time is not a constraint, KV is an easy-to-use and cost-effective video annotation software for 2D kinematic analysis (20). It offers many features for manual and frame-by-frame video editing and its reliability and validity have been assessed both in a clinical setting (21–23) and in sports performance (24–27).

In this study, the anatomical landmarks that had markers placed on them, were used to annotate the joint centers. For the hip joint, the center of rotation was placed approximately on the greater trochanter of the femur. It was mediolaterally defined at the center of the thigh and, in terms of height, across the line of the Iliac symphysis. The distal markers were on the shoulder and the knee. The rotation center of the hip did not had a marker on it, and it was defined based on the motion of the leg during walking. For the knee joint, the center of rotation was defined at the knee’s marker while the distal landmarks were the rotation center that was defined on the greater trochanter by the user and the malleolus marker. For the ankle joint, the center of rotation was placed on the malleolus. The other two defining markers were on the knee and at the 5th metatarsal. In order to get values comparable with the VC, an angle of $90^{\circ }$ was subtracted from the measurements and then the offset due to the person’s anatomy was removed. To do that, a static posture image was used to remove the offset when the leg was straight. A limitation of this process is that the use of markers as landmarks introduces bias to the manual annotation. There were three reasons why it was decided to continue the process despite of this. The first one is that different gait cycles recorded at different times will not be exactly the same. Therefore, there will be a variance that did not arise from the methods that were used. As such, the results would not allow for evaluation between them since the origin of the variance would be undetermined. The purpose of this work was to compare data that a professional could realistically obtain manually with pose estimation methods and quantify the inaccuracies. Therefore, the same signals were required across all systems. The second reason was that CGM 2.4 doesn’t use the markers to establish Joint Centers (JC) but to scale an Inverse Kinematic (IK) model. This means that the actual JCs are products of an optimization process and may not necessarily coincide with the markers. However, it is possible that the JC on the knee and the ankle will not be far from their markers, at the same time experts rarely have issues identifying these joints so the bias should be minimal. The hip joint was the only one that needed to be identified based on the motion that was exhibited and it is the joint that is elusive across all methods. Lastly, the markers were placed by a team of experts on bony landmarks. As such, by following them, the variability that could arise between successive annotations and different raters is reduced. It should be noted that assessing inter- and intra-rater variability was outside the scope of this work, so the focus was to minimize bias from random sources. Ultimately, because of the different prerequisites each method has, a process that balanced the different sources of bias in a realistic manner was followed.

### 2.5. Pose estimation with Openpose and Mediapipe

There are two main categories of pose estimation algorithms. The top-down algorithms and the bottom-up. Top-down methods identify a human as an object in an image, then the pose is determined. The problem with this approach is that if the initial identification fails, then there’s no way to recover (28). These methods tend to be less computationally intensive when only a single person is in the image. However, their demands increase when they attempt to identify multiple persons. Bottom-up approaches identify keypoints first, and then cluster them together to estimate the person’s movements. They can be more computationally demanding, but their cost doesn’t increase as a function of the number of people. However, the clustering may fail when there’s an overlap of body parts (28).

In this work, Openpose v1.7, which is a bottom-up method, and Mediapipe v0.9.0.1, a top-down algorithm, were employed. A comprehensive technical description of pose estimation algorithms is outside the scope of this paper. Suffice to say that OP is a multi-stage CNN that uses the first 10 layers of the “Visual Geometry Group” (VGG) architecture (29) to generate representative feature maps from the RGB input. Then, the first stage CNN uses the feature maps that were created to produce a set of Part Affinity Fields (PAFs) representing the association of the detected parts in the input image. The second stage is responsible for estimating part confidence maps, i.e., the level of confidence regarding the association of different PAFs. The output of each convolutional layer in a single-stage CNN is concatenated in the final output, adopting a technique proposed in DenseNets (30). Based on those two maps the final output is attached to anatomical landmarks based on the pose model that is used. On the other hand, MediaPipe uses a variation of the lightweight, well-established MobileNetV2 model (31) to detect the presence of bodies in the input RGB image in real time. This is done using a set of a few pose keypoints. Then, a real-time body pose tracker (32), a generative model for 3D human shapes and poses named GHUM (33) are applied. Finally, a pose estimation with 33 landmarks in 3D is produced.

OpenPose supports two pose models: BODY_25 and COCO. The relevant difference that these two models have is that BODY_25 has keypoints on the foot, which allows for measurements on the ankle, while COCO ends at the ankle joint. Therefore, BODY_25 is the model that is used in this work. A simple custom script in Powershell was written for OP to extract the keypoints as .json files (one file for each frame). The .json files were organized into folders for each subject and were later processed using scripts in Matlab to organize the output into a format that could be easily manipulated.

Mediapipe had a better interface and it was quite simple to write a script in Python that would extract all the keypoints into a single Python dictionary. However, it was decided to perform all calculations in Matlab for logistical reasons. Therefore the raw output from both pose estimation algorithms (the .json files and the python dictionaries) was processed by the same Matlab script.

### 2.6. Statistical analyses

The ground truth was the output of VC. Joint angles that were measured from the marker-based system were compared against all other methods. Evaluating different methods has certain caveats that need to be considered in order to avoid Type I errors. The primary source of false positives is that even though the data are being measured using different approaches, they have to be the same to ensure that whatever variance is detected originates from the method.

As such, the correlation coefficient R is inherently high regardless of the actual agreement between the methods. In general, when comparing methodologies, the question shouldn’t be how much they agree, but how much they differ and if that difference is significant (34). Another important aspect is the output of each method across a range of measured values. This helps to identify cases where the accuracy diminishes even if the overall behavior is still within acceptable limits of difference. Indeed, extracting a single value such as the coefficient R or the t statistic, oversimplifies vector trajectories and may give a false impression of the accuracy of each method.

In this work, the evaluation of the different methods is done using the statistical parametric mapping (SPM) and the Bland-Altman (B-A) difference against the mean graph. Initially used for neuroimaging analyses (35) and later adapted to biomechanics (36), SPM considers covariance among vector components. Mean continua are extracted from the measurements of each method and the variance is studied. The point of relevance here, is that regions of the joint angles that are not adequately accurate are highlighted, allowing for a granular assessment of the AI methods. This can direct efforts of enhancing the pose estimation algorithm for the problematic cases. The SPM calculations were done using the SPM1D API (spm1d.org).

Complementary to SPM, the evaluation of the differences between methods was also done using B-A graphs (34,37) to have an overview. A mean gait cycle from each method was used for the B-A graphs, using more gait cycles would clutter the graphs obscuring the pattern. This is why B-A is used alongside the SPM, to have both a detailed behavior and a general description. The difference in the mean error between each method was plotted against the mean error in a scatter plot. This method can show not only the average error between the two methods, but also how clustered the data points are around the error. The limits of the confidence intervals (CI) were set as ±1.96  *  standard deviation (37), however, attention was given to the range of the CI as well. In essence, it was possible to see how wide the measurements are scattered around the mean error.

## 3. Results

In this section, the comparison between VC and the other three methods is shown first. Then, the comparison between OP and KV is presented. This is done to explore if these two systems can complement each other and if accurate results can be achieved without access to a marker-based system. It should also be noted that all images are present in the Supplementary Material at their full size for clarity, along with a few, somewhat redundant but possibly interesting, extra graphs of the repeated SPM ANOVA for VC, KV, and OP.

### 3.1. Vicon vs. Kinovea

**Figure 3:**
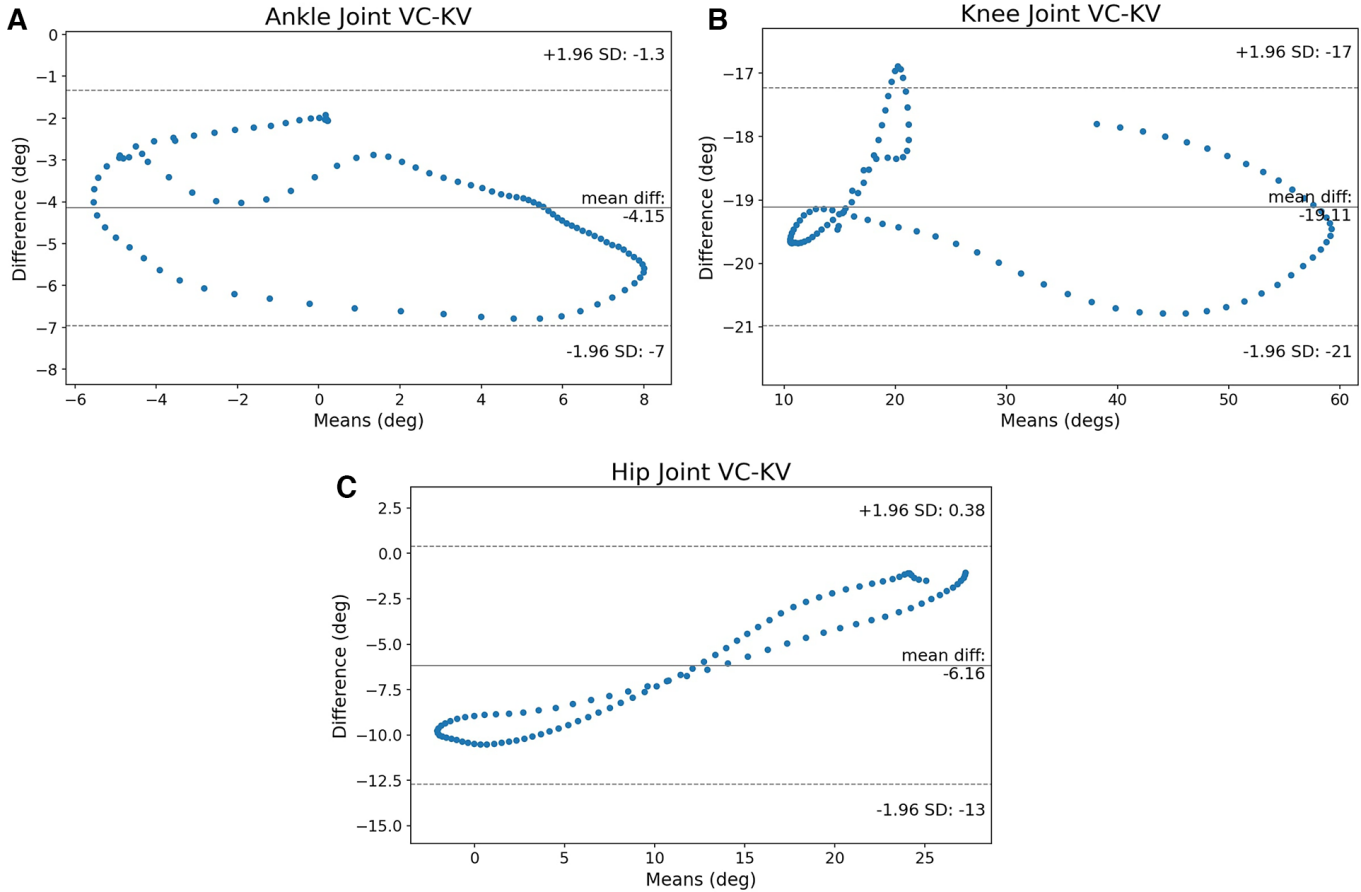
The B-A graphs between VC and KV for ankle, knee, and hip joints. The ankle joint has many values that are close to the CI and the knee crosses the limits, the hip joint’s values are not clustered around the mean, but the error appears to have a pattern. (**A**) B-A graph of the ankle, (**B**) B-A graph of the knee and (**C**) B-A graph of the hip joint.

The first metric that needs to be examined is the B-A graphs between VC and KV. Figures 3A–C show the B-A scatter plots for ankle, knee, and hip respectively.

The ankle joint in Figure 3A appears to have a lot of measurements near the CI limits but they never cross the lines of significance. Figure 3B shows that it is possible for KV to measure angles that are outside the CI. In general, even though the majority of measurements are clustered near the mean difference, there is also a portion of joint angles that are near or outside of the CI. Figure 3C shows that on the hip, the measurements tend to be on the left-bottom and right-top parts of the B-A graph. It is also worth noting that the CI is $13.38^{\circ }$, the largest of the three joints.

**Figure 4:**
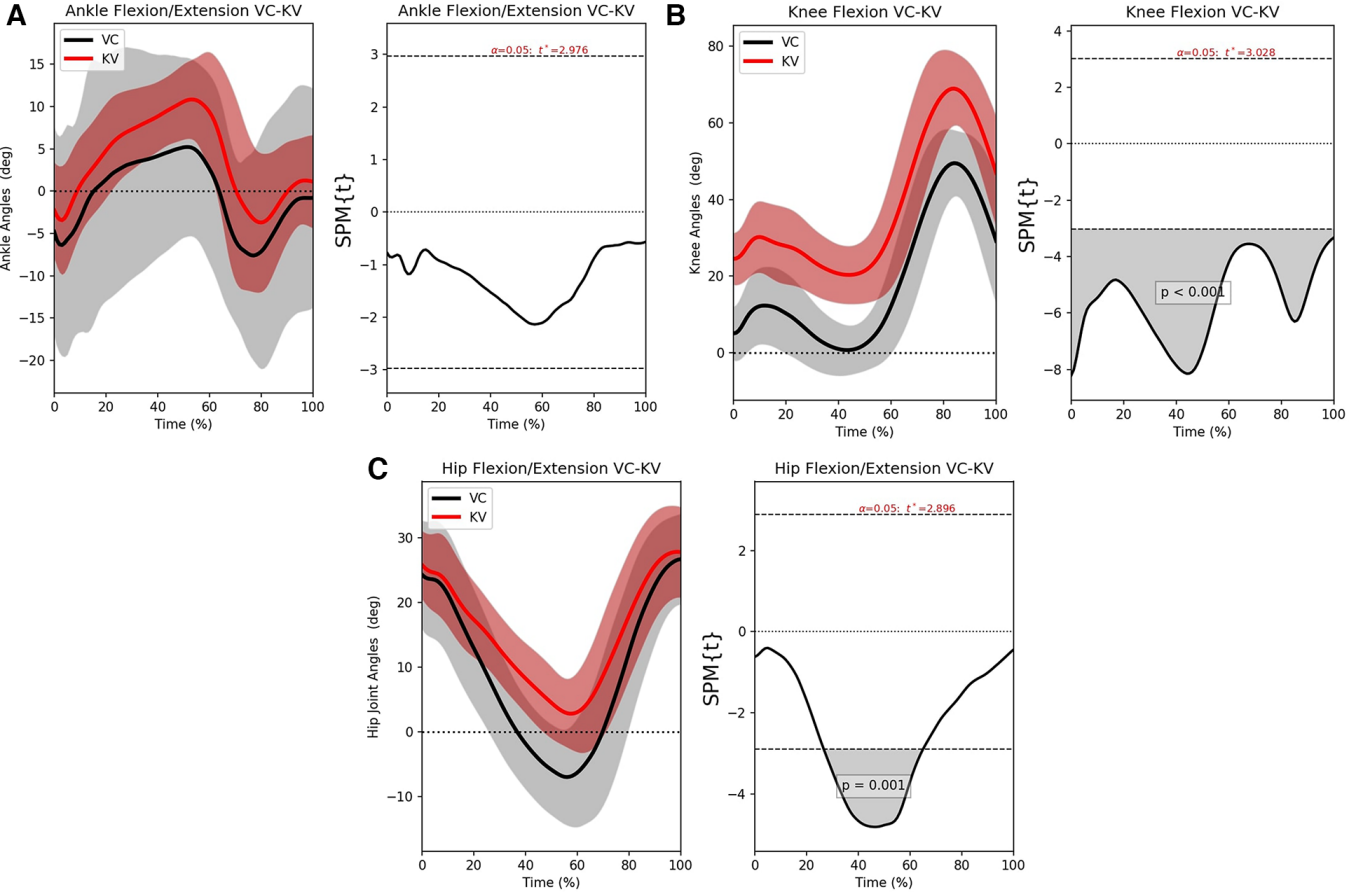
The left-hand graphs of the figures shows the mean motion waveform with their respective standard deviations. The right side shows the statistical significance of the error, wherever the area under the curve is greyed out, there is significant difference. (**A**) SPM analysis for the ankle joint, (**B**) SPM analysis for the knee joint and (**C**) SPM analysis for the hip joint.

For the ankle, knee, and hip joints, the SPM mean continua analyses are examined next to identify the problematic areas. Figures 4A–C shows the statistical parametric mapping between VC and KV for the three joints.

Observing Figure 4A, it can be seen that the error between VC and KV is consistent but well away from significance. It is also interesting to see that the variance of the VC (grey area) is larger, while the KV variance (red area) is more focused around the mean motion. This means that the user was more consistent but less accurate.

From Figure 4B, KV has a relatively stationary offset of about $20^{\circ }$ when compared to VC. According to the SPM analysis, there is significant disagreement for all of the gait cycles but the pattern is preserved. This is because identifying the hip joint center is challenging, especially during the terminal stance and pre-swing phases of gait. As a result, the user’s bias will add an offset to the knee joint angles that is more pronounced around 30%−−50% of the gait cycle. However, the pattern and the range will still be preserved, which makes it possible to account for that offset.

Figure 4C shows that the hip also has an offset between the two methods and the statistically significant region is at the middle of the cycle. As before, the issue is that the hip joint is difficult to identify visually with KV and its motion might be underestimated. Referencing back to the B-A graphs (Figures 3B,C), the cases crossing the CI on the knee and the large CI on the hip are justified since they are affected by the placement of the hip joint center. As such, it is not surprising that the SPM shows error when the leg is perpendicular to the ground and the hip center of rotation cannot be distinguished.

In general, manually annotating the joints will preserve the motion similar to a marker-based system but in certain situations, the error of the user will introduce a significant offset. What is important to note, however, is that the KV user will be consistent.

### 3.2. Vicon vs. OpenPose

**Figure 5:**
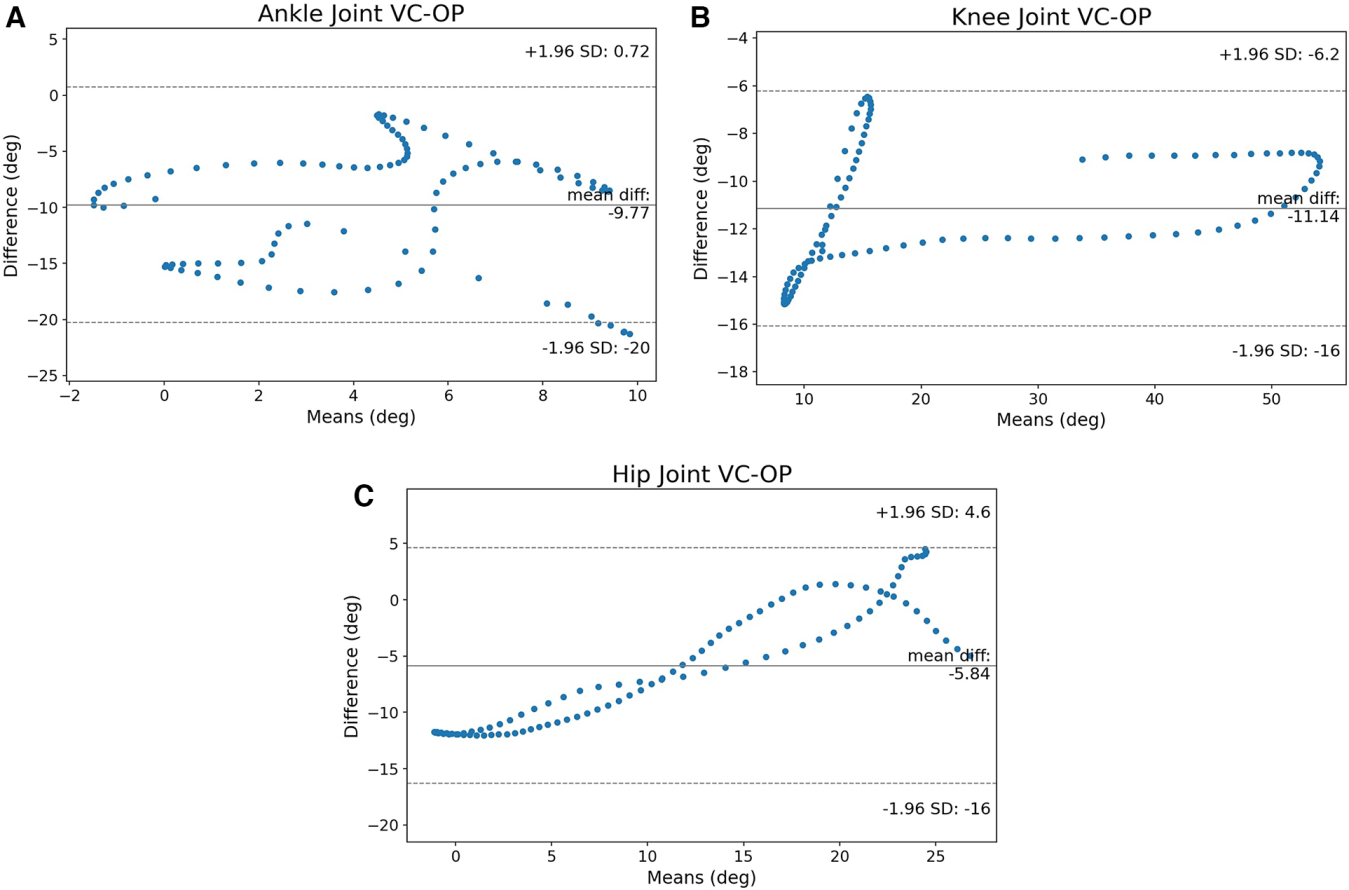
The B-A graphs between VC and OP for ankle, knee, and hip joints. The ankle joint appears erratic, the knee approaches the limits in certain regions of the gait cycle. The hip joint’s values are not clustered around the mean but the error appears to have a pattern. (**A**) B-A graph of the ankle joint, (**B**) B-A graph of the knee joint and (**C**) B-A graph of the hip joint.

The B-A graphs between VC and OP are examined in this section. Figure 5A shows the ankle joint angles. There is only a small portion of measurements that appear to be significantly different between the two methods. However, the distribution appears to be somewhat erratic in Figure 5A with certain values crossing the CI limits. Figure 5B shows that the knee joint angles are clustered around the mean with a very narrow CI, while the hip joint in Figure 5C has the CI of $20.6^{\circ }$ similar to the ankle joint ($20.76^{\circ }$). Both knee and hip do not cross the CI limits though that might not be the full story. The SPM analyses highlight the regions of interest.

**Figure 6:**
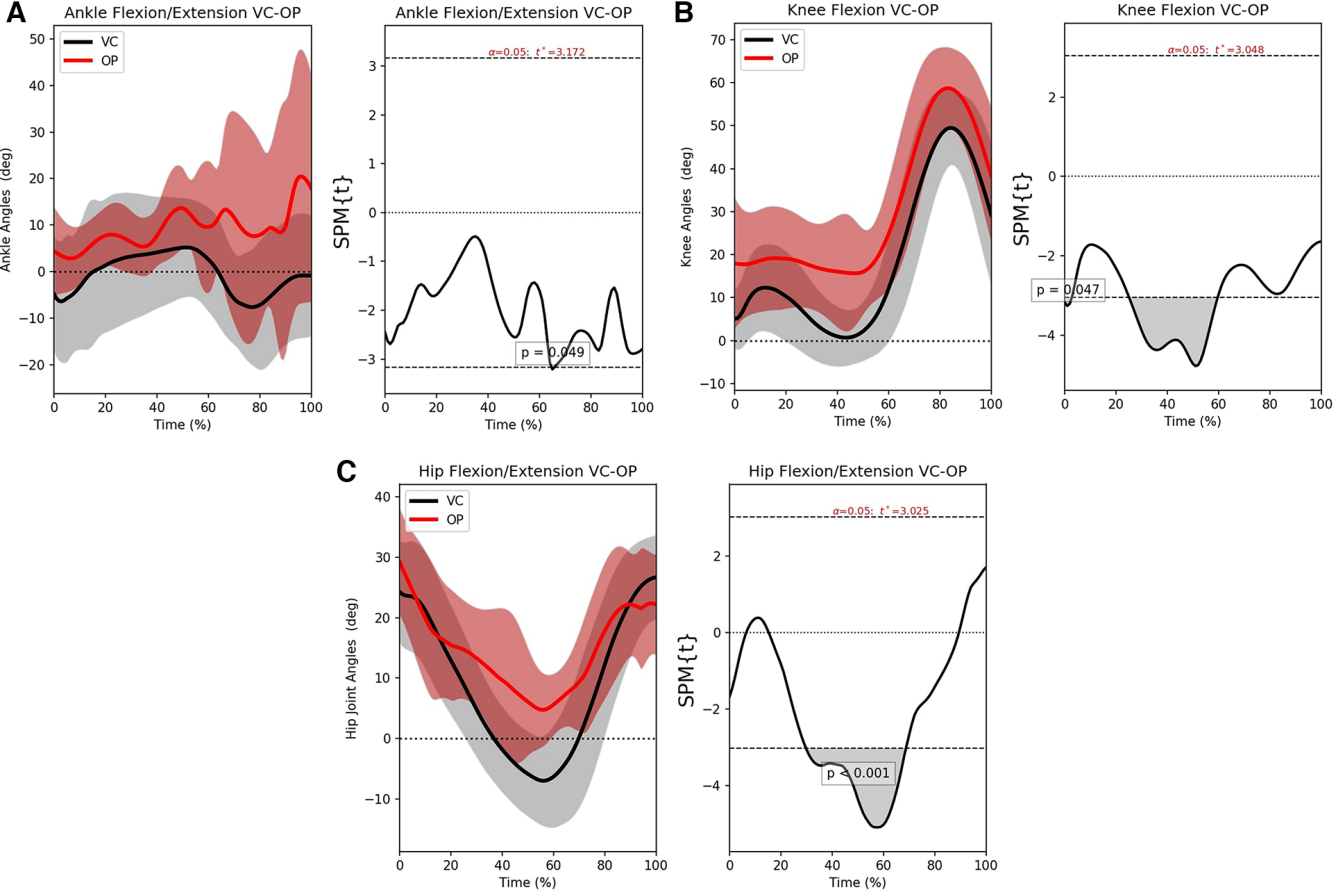
The left-hand graphs of the figures shows the mean motion waveform with their respective standard deviations. The right side shows the statistical significance of the error, wherever the area under the curve is greyed out, there is significant difference. (**A**) SPM analysis for the ankle joint, (**B**) SPM analysis for the knee joint and (**C**) SPM analysis for the hip joint.

The mean motion of the ankle from OP in Figure 6A reveals how unstable OP is in tracking the foot. Indeed, visual examination of the videos shows the OP keypoints on the metatarsals to be misidentified on multiple frames. Though the error is not statistically significant, this is a Type I error (false positive), since OP fails to follow the overarching pattern of VC. The large variance (red area) in Figure 6A is also a sign of erroneous tracking of the metatarsals

Figure 6B shows the opposite behavior. The knee joint angles are measured accurately with OP. The significant error occurs in the middle of the gait cycle. This is again an error arising from different identification of the hip joint center between the systems. It should be noted though that OP has a smaller offset than KV. The knee has the highest range of motion and according to the B-A graph and the SPM analysis, it is the joint that is being tracked the best. Finally, the hip joint in Figure 6C shows that OP suffers in the same areas as KV but the JC is tracked better.

In general, OP can have impressive accuracy for the correct joints. However, it is more sensitive to misplacing the keypoints and this will have consequences. It is also highlighted that this is a problem that affects joints that exhibit low motion since even the knee and the hip can have larger errors when they are relatively stationary. That said, the agreement of OP with VC is still impressive and the potential to become an accessible tool for gait analyses is a realistic possibility.

### 3.3. Vicon vs. Mediapipe

**Figure 7:**
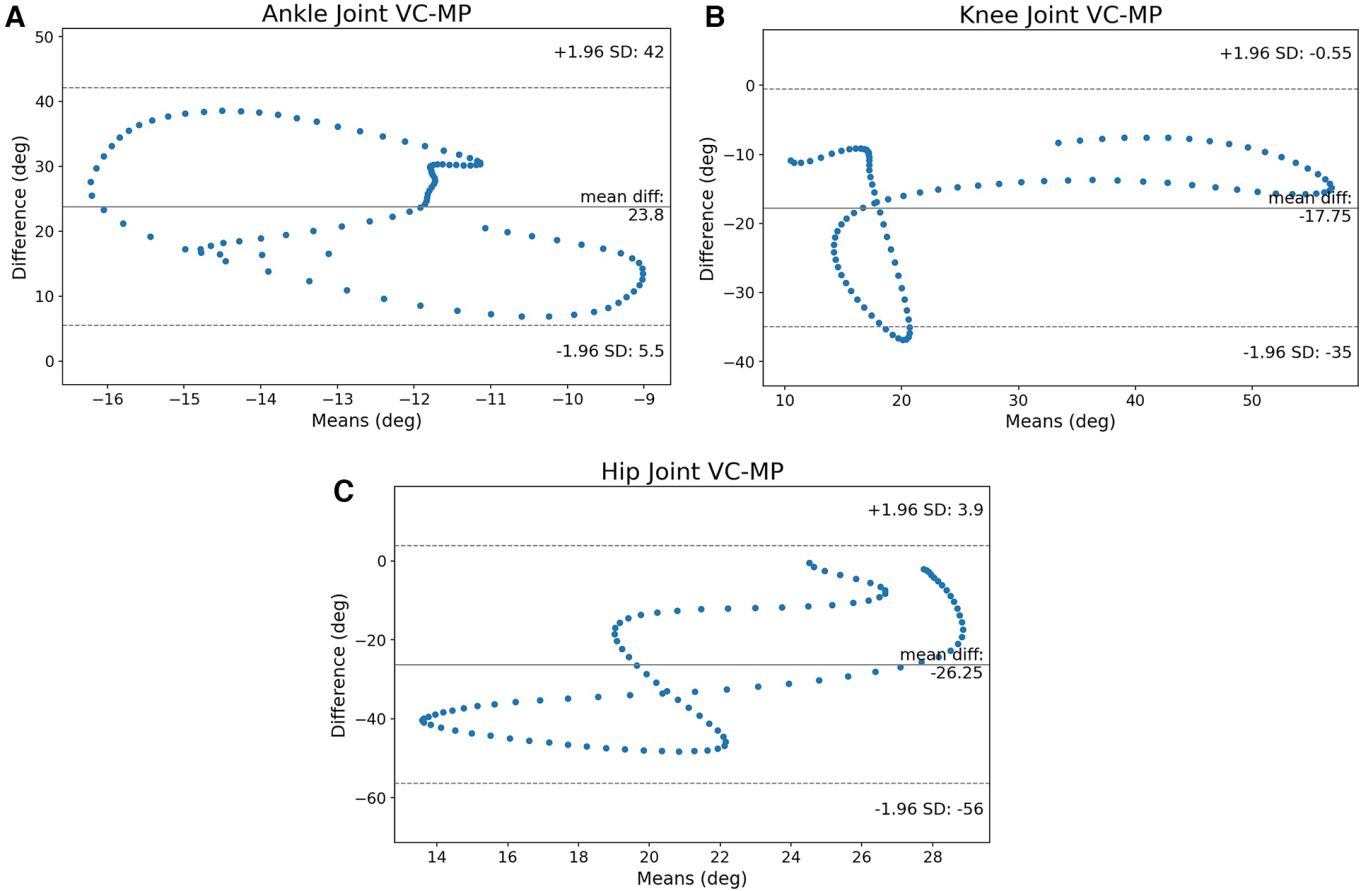
The B-A graphs between VC and OP for ankle, knee, and hip joints. The ankle joint is very spread out, the knee crosses the limits in certain regions of the gait cycle. The hip joint’s values are not clustered around the mean. (**A**) B-A graph of the ankle joint, (**B**) B-A graph of the knee joint and (**C**) B-A graph of the hip joint.

The B-A graphs for the three joints of interest are shown in Figures 7A–C.

The most striking observation is that the range of the CI is pretty large for all joints compared to the other methods. Indeed, even the knee that had the smallest CI so far, shows a large confidence interval in Figure 7B. The uniform distribution of the data points on the whole region of the CI shows that the error is very spread out around the mean value. Therefore the accuracy is greatly diminished throughout the gait cycle.

**Figure 8:**
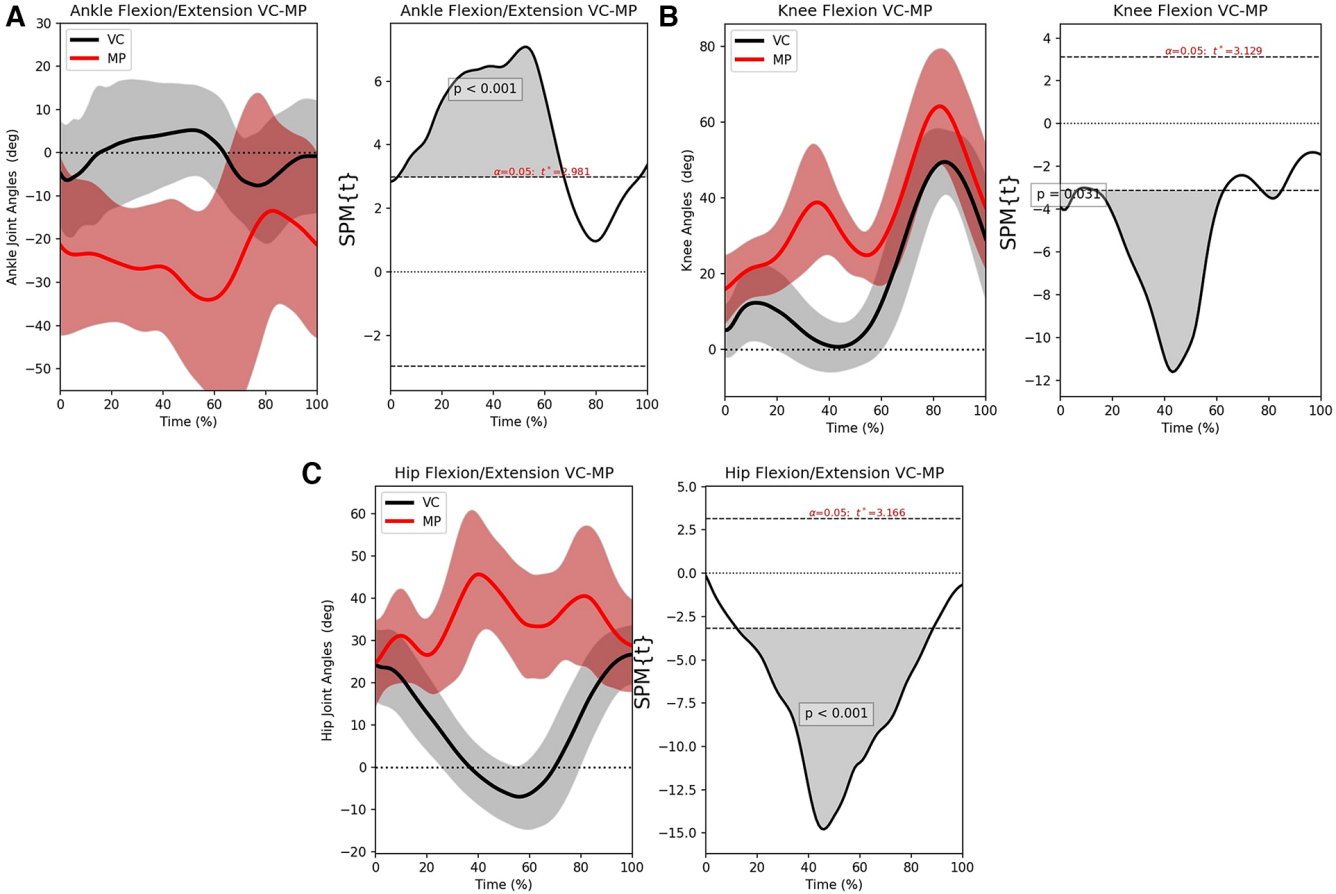
The left-hand graphs of the figures shows the mean motion waveform with their respective standard deviations. The right side shows the statistical significance of the error, wherever the area under the curve is greyed out, there is significant difference. (**A**) SPM analysis for the ankle joint, (**B**) SPM analysis for the knee joint and (**C**) SPM analysis for the hip joint.

The SPM analyses show that the results are as problematic as the B-A graphs suggested. The ankle joint is completely misidentified as shown in Figure 8A. Similarly, Figure 8C shows that the hip suffers as well. MP seems to struggle to separate the left and right sides and that has led to large variances (red area). The clearest example is the knee SPM analysis in Figure 8B that shows a surprising agreement during flexion. However, during the stance phase, there is a motion recorded across all subjects. This happens because the algorithm mislabels the left and right knee when the right leg crosses from behind the body to the front. Indeed, visual inspection of the videos showed that MP’s skeleton was flickering and mislabelling joints a lot more than OP’s. This is the source of the error that produced the results presented here.

### 3.4. Openpose vs. Kinovea

From the previous subsections, it is clear that OP and KV can be fairly accurate and can potentially complement each other. To explore this, the OP and the KV results are compared. It should be stressed that, unlike the previous sections, it is not the agreement of the methods to the ground truth that is examined, but the agreement with each other.

**Figure 9:**
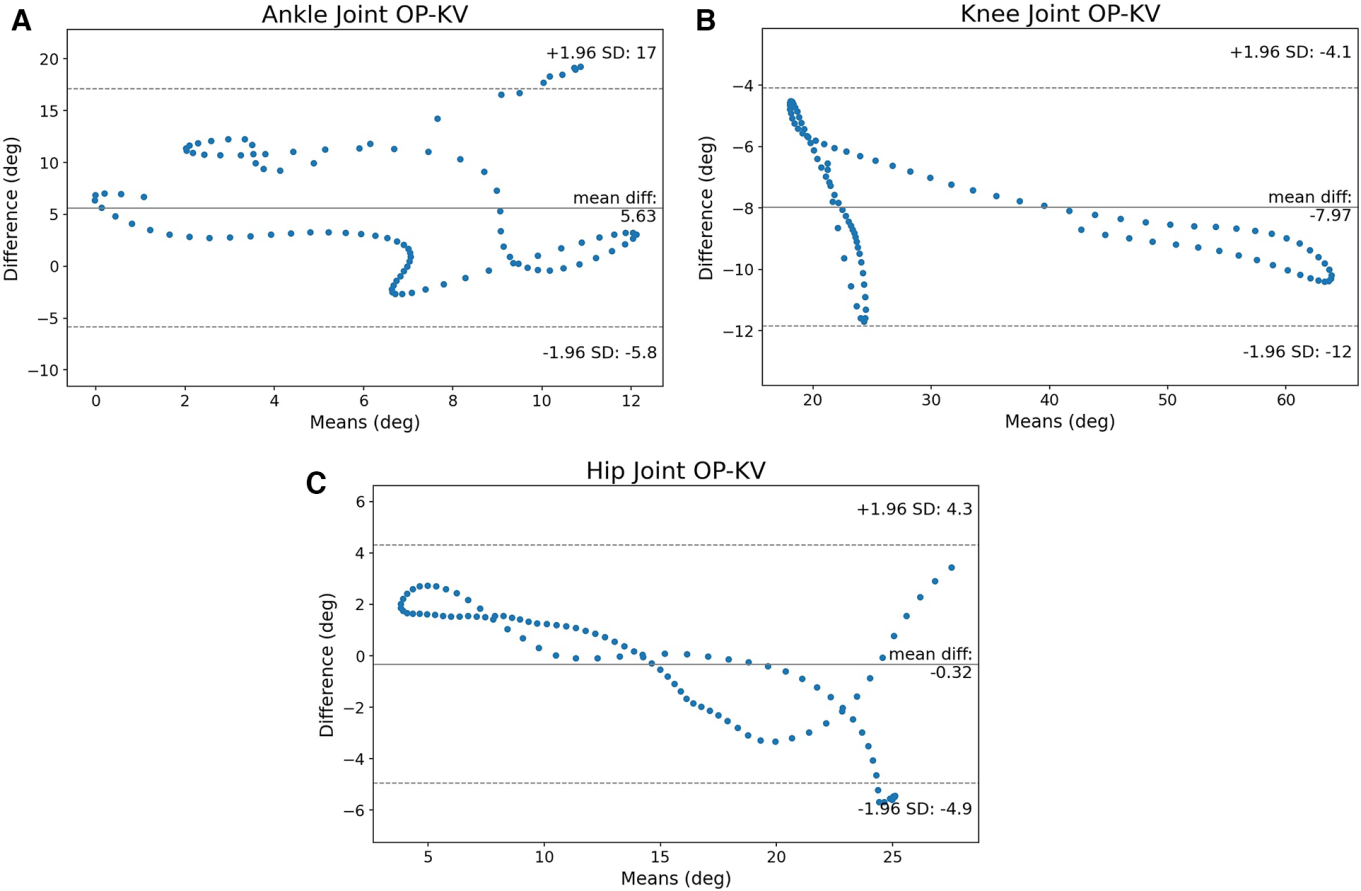
The B-A graphs between OP and KV for ankle, knee, and hip joints. The ankle joint appears erratic, the knee approaches the limits in certain regions of the gait cycle. The hip joint’s values are clustered around the mean, but they cross the CI for a small number of cases. (**A**) B-A graph of the ankle joint, (**B**) B-A graph of the knee joint and (**C**) B-A graph of the hip joint.

Figure 9A shows a rather wide CI with the values scattered throughout the whole region and some values crossing the limit. This was expected since OP failed to follow the ankle. The agreement is much better on the knee, as Figure 9B shows. The mean error is lower than when either method is compared to VC with the CI being larger from VC-KV but smaller from VC-OP. The SPM analysis gives a better insight of both methods track. Finally, in Figure 9C, the hip exhibits a very high agreement with a low mean error and the measurements clustered around it. However, some values cross the CI limit and a closer examination is in order.

**Figure 10:**
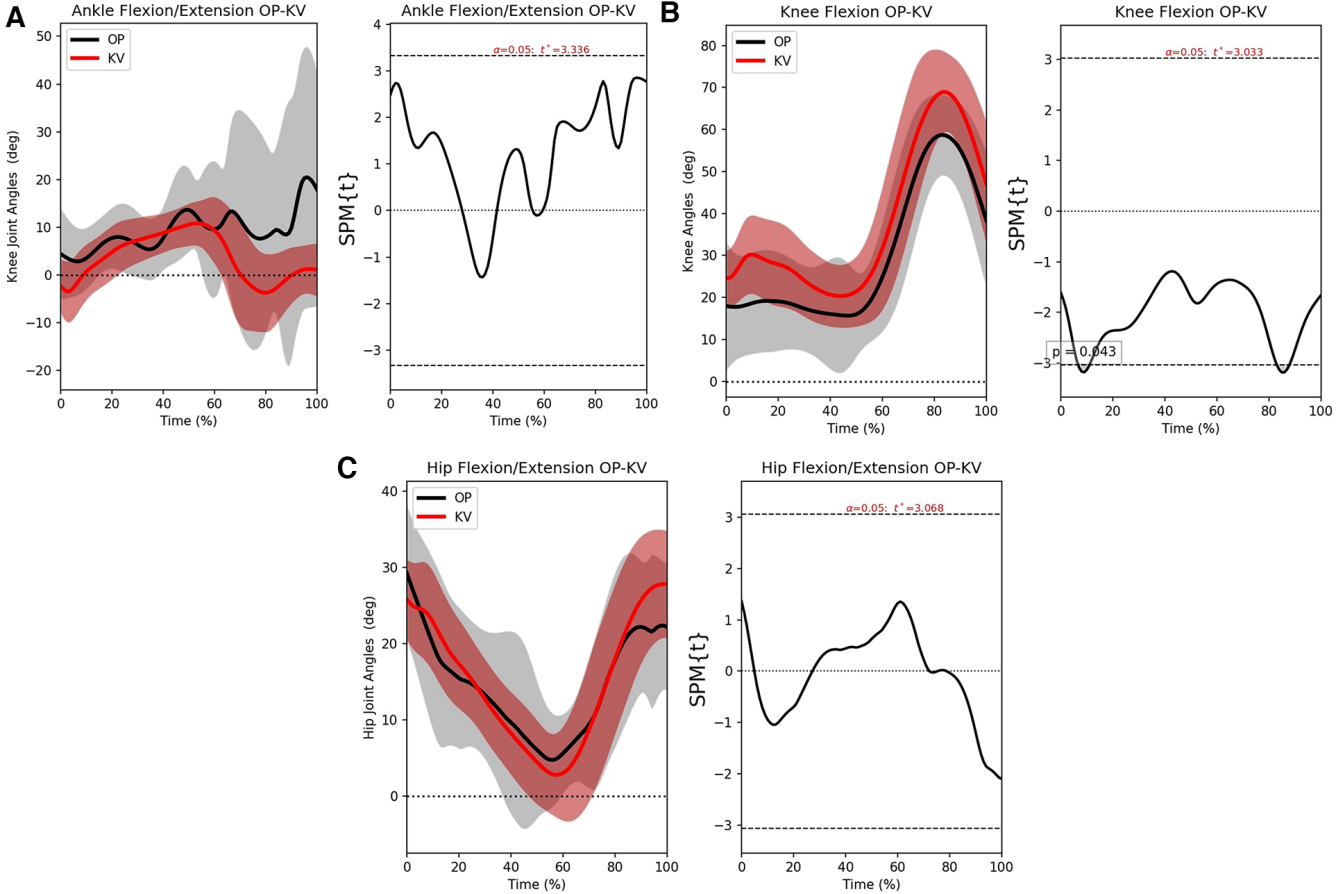
The left-hand graphs of the figures shows the mean motion waveform with their respective standard deviations. The right side shows the statistical significance of the error, wherever the area under the curve is greyed out, there is significant difference. (**A**) SPM analysis for the ankle joint, (**B**) SPM analysis for the knee joint and (**C**) SPM analysis for the hip joint.

Figure 10A is very similar to Figure 6A since the erratic behavior of OP on the ankle renders the results inaccurate. Figure 10B is more interesting because it shows that the offset of the KV pushes the error slightly above significance (below the confidence interval’s lower limit) twice. However, it becomes insignificant again quickly and remains like that. Finally, Figure 10C, shows that the hip joint can be confidently measured with either KV or OP and have the same results. Please note that this simply means that the hip will have equivalent error regardless of the method, rather than it is measured more accurately.

### 3.5. Time of processing

Using KV, each gait cycle took about 2 to 2.5 h to get the final measurements. That’s roughly 38 h for all 17 subjects. This was by far the most time-consuming and taxing method with no practical way to speed it up. Pose estimation algorithms were on a different order of magnitude. It took about 2 minutes and 9 s to run all 17 trials in OP, while MP required 87 s to process the videos.

## 4. Discussion

In this work, a multicamera marker-based system for motion capture that has become the de facto standard (VC) was compared with three open-source methods to calculate the leg’s joint angles during a gait cycle. Those were the Kinovea video annotation tool, the Openpose pose estimation algorithm, and the Mediapipe pose estimation algorithm. The goal was to examine if it is possible to get accurate data using more accessible tools. The B-A graph was used to examine the degree of difference between each method and the SPM t-test was employed to identify the phases of disagreement during gait.

Results show that MP had the worst output of all tested methods. However, as Figure 8B shows, when the motion was sufficiently large and the joint is unambiguously discernible like knee is, it can produce very accurate results. This is probably a result of MP’s top-down approach. As such, despite being less computationally expensive, it is not recommended for gait analysis.

KV showed that a static offset, the bias of the user, was present throughout the recording, but it was consistent for the majority of the measurements. The source of the error was the difficulty to identify the hip joint center. It can be argued that a more standardized approach to identify the joint center, such as adding a marker (not necessarily reflective) or review from multiple users can alleviate the issue. However, this will not solve the issue that KV is extremely dependant on human input. Since the time investment that it requires from the person who performs the analysis is very high, adding more users to the workflow will have diminishing returns.

Pose estimation using OP had the most promising results but some caveats need to be considered. The ankle suffered from keypoint misplacement, it didn’t help that the joint itself exhibited very little motion. Even though there wasn’t a significant error, this was a Type I error because the overarching pattern of the ankle wasn’t preserved. This particular joint is extremely important for biomechanic analyses during gait but it is elusive to measure using pose estimation methods. The knee is the most successful result of OP. This is because the joint center is relatively easy to identify, the segments attached to it are substantially long and rigid, and it exhibits the largest range of motion during gait. However, hip misidentification still affected it. Despite that, knee flexion is the safest motion to use OP. Interestingly, the hip exhibits significant error at the same phases as the KV results. This is not surprising considering that OP used videos annotated by humans to train its AI model. The implication here is that human bias is part of the OP’s algorithm.

The bias that has affected OP’s training is apparent in subsection 3.4. The ankle wasn’t measured accurately, so there are no safe conclusions to be drawn, but the SPM analysis shows that the OP tracking does not deteriorate completely up until 60% of the gait cycle. This is perhaps grounds for future investigation. The knee, once again, is a great example because it seems that the bias between OP and KV is very close to the bias between VC and KV. This was expected because OP manages to follow VC very closely, thus Figure 10B is very similar to Figure 4B. Since the knee is the easiest joint to identify, it was possible for the AI system to achieve high accuracy from the training dataset. Lastly, the hip joint had an impressive agreement between the two methods. Considering that VC uses its own algorithm of inverse kinematics to define the hip joint center, and OP uses a large dataset that was annotated by humans, it becomes apparent that OP has achieved a high level of mimicry, but it also means that the error of the dataset has been transferred as well.

The lower computational demands of MP came at the cost of reduced accuracy to such an extent that it cannot be used for gait analyses in its current iteration. On the other hand, in agreement with the literature, OP appeared to be able to correctly identify and follow the joints for the majority of the motions examined, albeit it was more taxing on the hardware. Manual annotation using KV is still a viable option as long as the user’s bias is taken into account. It should be noted that the time required for a single trial makes it difficult for larger scale studies. However, the low cost, and the potential for a single professional to be able to get data on par with a marker-based system makes it appealing to try to work around the issues surrounding KV and OP.

The time difference between MP (87 s), OP (129 s) and KV (≈38 h) cannot be ignored. The most obvious solution would be a video annotation tool that would integrate OP, or perhaps MP for lower-end computers, and would allow manual corrections on the output. This would reduce the time to annotate a video and its accuracy would be comparable to a multi-camera system.

It should also be understood that AI pose estimation accuracy hinges on the the quality of the labelled training dataset. This has been pointed out in the literature (1) but after comparing the VC with KV (Figure 4C), VC with OP (Figure 6A) and OP with KV (Figure 10C), it would seem that the human bias has leaked into the trained model and it is a powerful source of error. As such, videos annotated by a marker-based system might be more appropriate to train pose estimation algorithms in the future.

## 5. Conclusions

In conclusion, OP is superior to MP despite the higher computational cost. However, it suffers on certain joints and when the range of motion is not large enough. Manual annotation with KV has an offset but it’s consistent and accurate. More importantly, KV allows for the user to fine-tune the keypoints in case of problematic recordings. From the results presented here, the pose estimation algorithms are accurate enough but they lack flexibility. Though the creation of better AI pose estimation systems is a very active field of research, the practical problem is that if the automated system miscalculates, the user has no way to intervene. This can become an even bigger problem if people with movement disabilities (prosthesis users, cerebral palsy, etc.) are measured with such systems. If markerless pose estimation is to become a part of the standard practice for health professionals, a platform that will use such systems and then allow for manual editing appears to be the most viable strategy in the near future.

## Data Availability

The raw data supporting the conclusions of this article will be made available by the authors, without undue reservation.
