# Supplementary material for: Gait analysis comparison between manual marking, 2D pose estimation algorithms, and 3D marker-based system
Source: Front Rehabil Sci. 2023 Sep 6;4:1238134. doi: 10.3389/fresc.2023.1238134 (PMC10511642; doi:10.3389/fresc.2023.1238134)
Supplement: Supplementary file 1 [file Datasheet1.pdf]

Supplementary Material

1 SPM REPEATED ANOVA BETWEEN VC, OP, AND KV

The statistical parametric mapping (SPM) anova comparison between VC, KV, and OP is shown in figure S1 through figure S3. It should be noted that MP has been excluded because of its high error that obstructed regions of agreement between the other methods. The ankle flexion/extension (figure S1) is a somewhat misleading graph because even though OP doesn't follow the pattern of the ankle joint, the range of motion is not large enough to produce a significant error when multiple methods are assessed. On the other hand the knee flexion (figure S2) suffers from the opposite problem. Because of the larger offset on the knee, the interaction between the variance of the three methods gives the impression that there's disagreement on the joint. The problem here is that any attempt to reduce the results may have an unforeseen impact on the statistical relationship. This was also the motivation to disqualify the R coefficient. Figure S3 shows is a more accurate representation of the hip's measurements. The figures here were excluded from the initial manuscript for brevity.

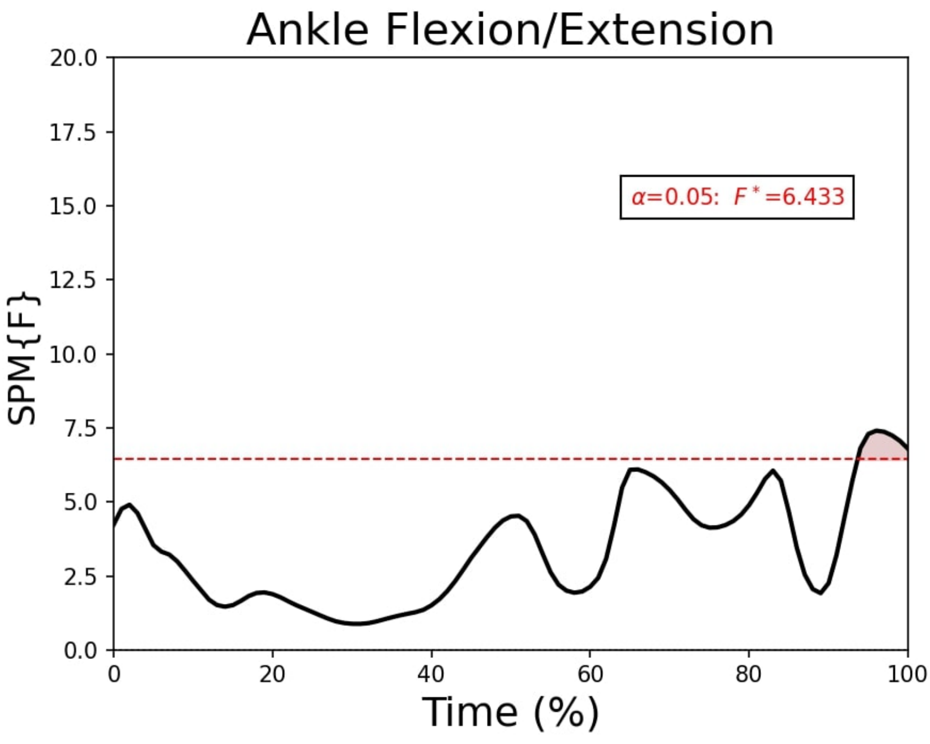

Figure S1. SPM comparison between VC, KV, and OP for the ankle

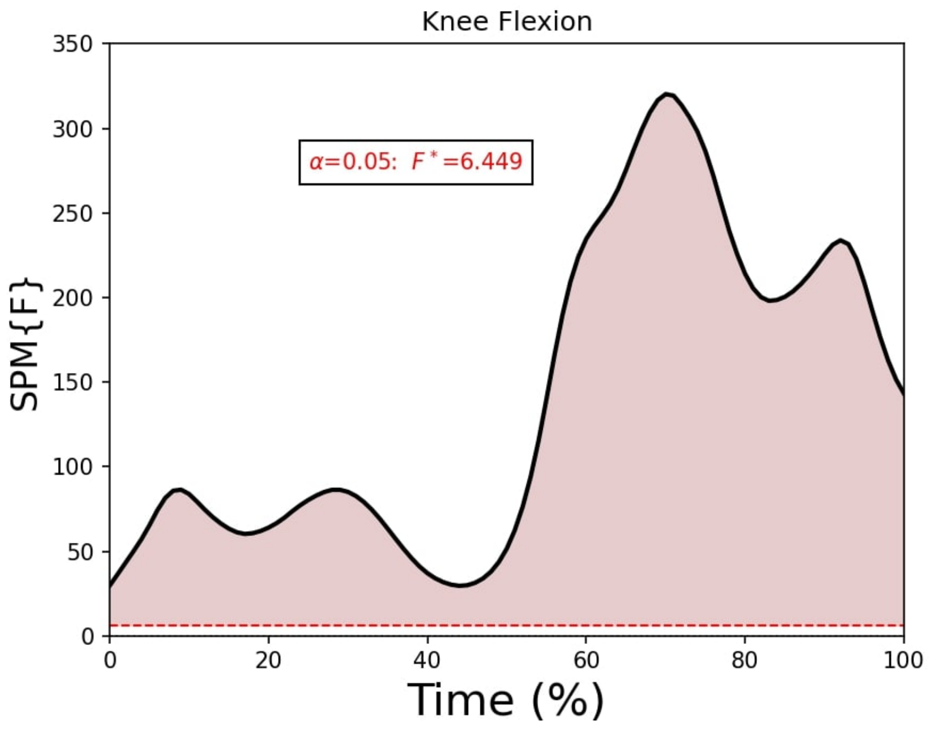

Figure S2. SPM comparison between VC, KV, and OP for the knee

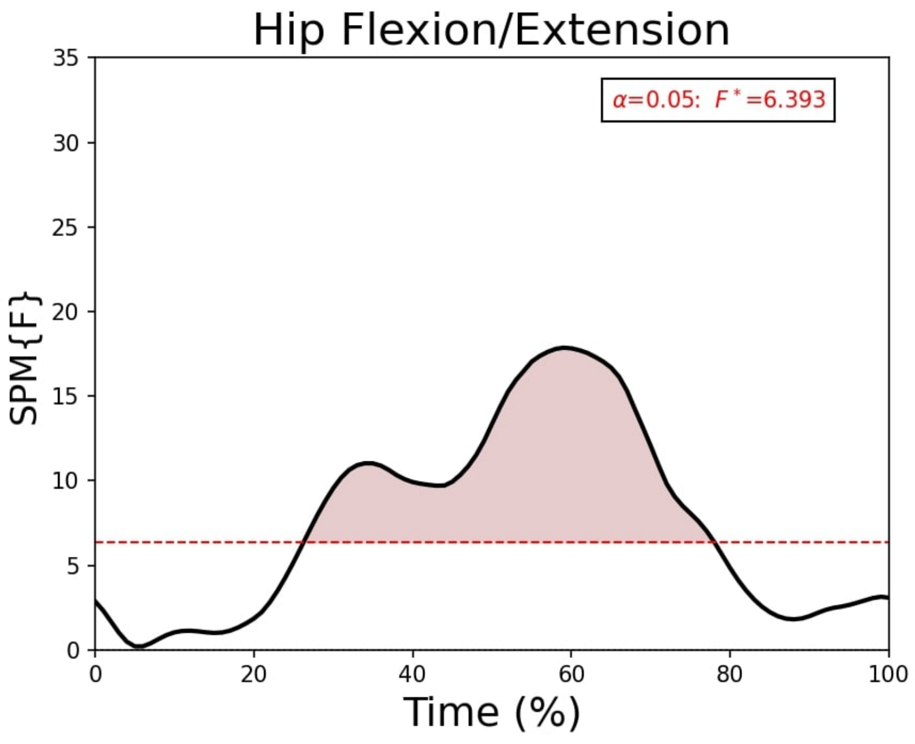

Figure S3. SPM comparison between VC, KV, and OP for the hip

1.1 Vicon VS Kinovea SPM

The statistical parametric mapping between VC and KV is given here. The figures are presented here at their full size for clarity.

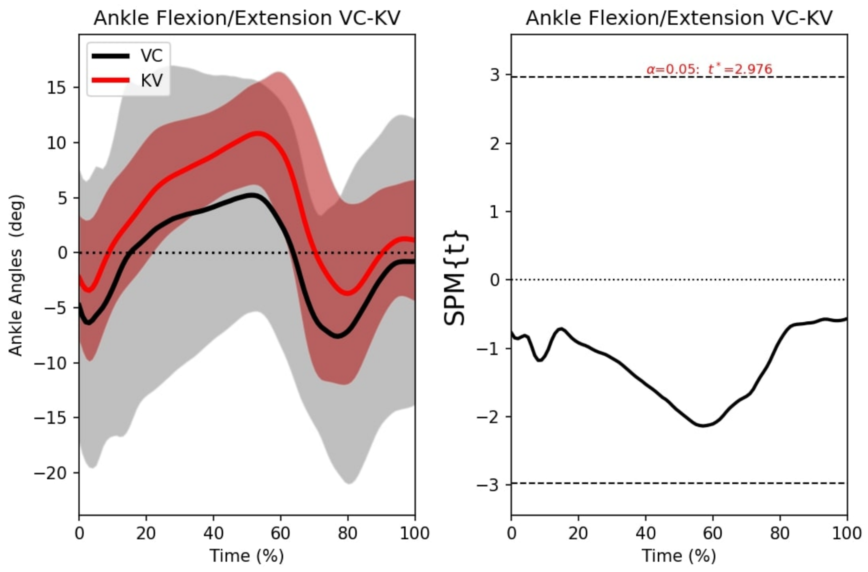

Figure S4. Statistical Parametric Mapping of the Ankle Joint Between VC and KV

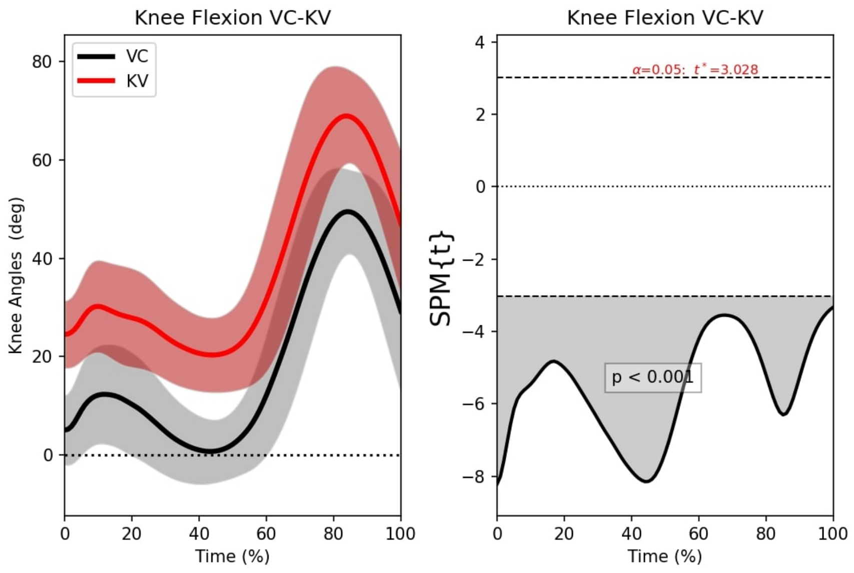

Figure S5. Statistical Parametric Mapping of the Knee Joint Between VC and KV

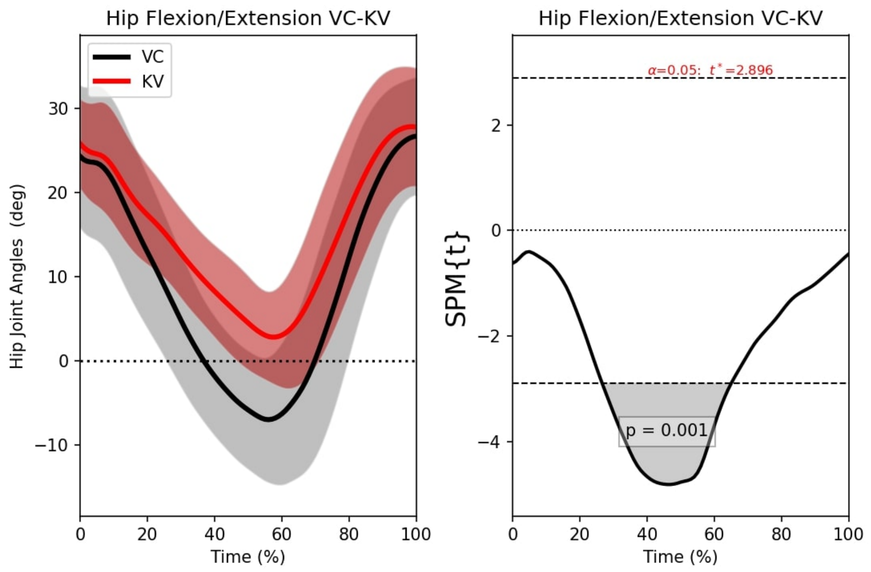

Figure S6. Statistical Parametric Mapping of the Hip Joint Between VC and KV

1.2 Vicon VS OpenPose SPM

The statistical parametric mapping between VC and OP is given here. The figures are presented here at their full size for clarity.

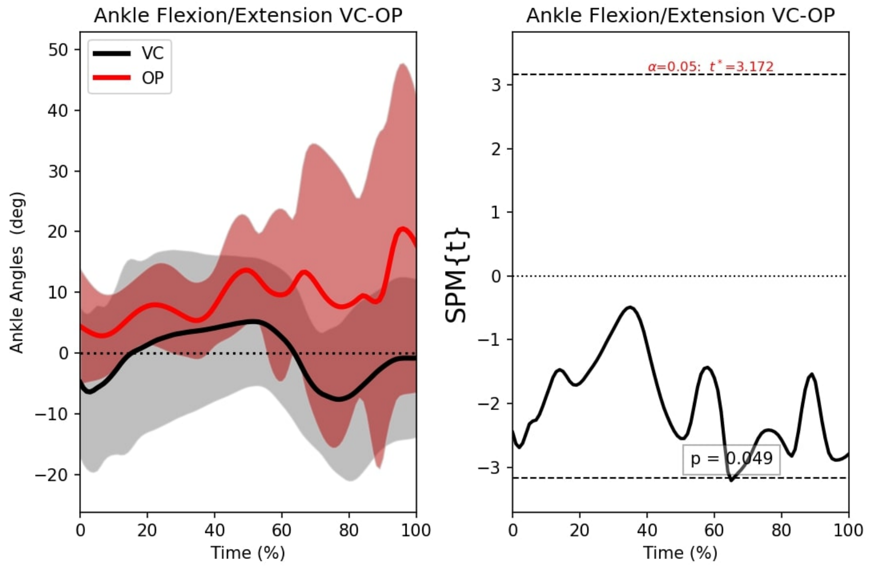

Figure S7. Statistical Parametric Mapping of the Ankle Joint Between VC and OP

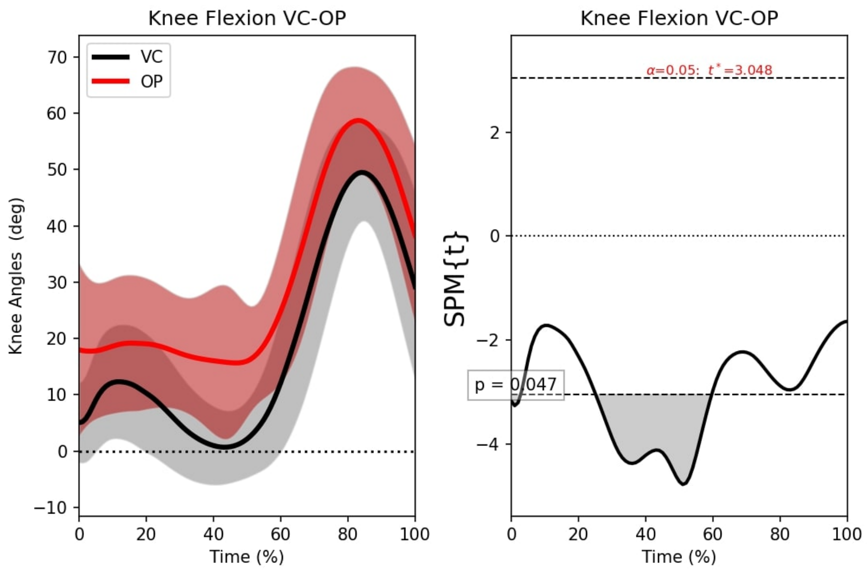

Figure S8. Statistical Parametric Mapping of the Knee Joint Between VC and OP

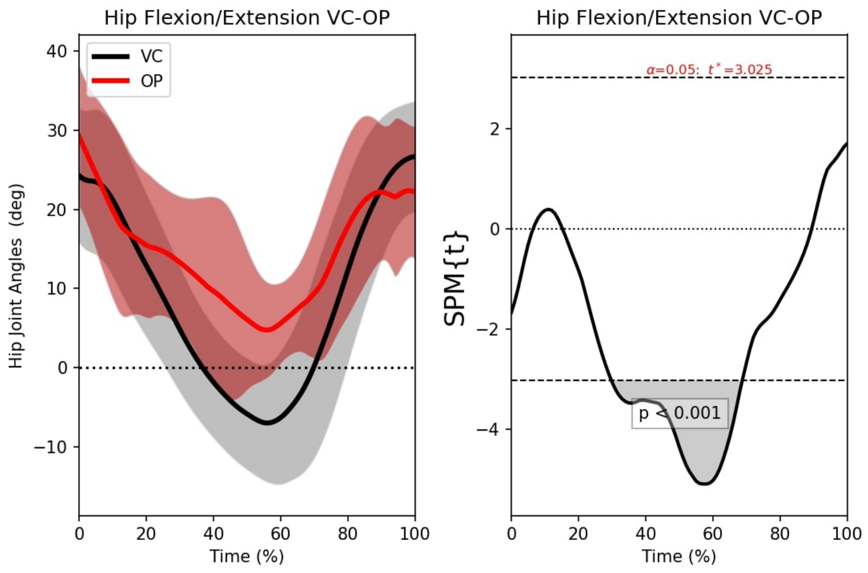

Figure S9. Statistical Parametric Mapping of the Hip Joint Between VC and OP

1.3 Vicon VS MediaPipe SPM

The statistical parametric mapping between VC and MP is given here. The figures are presented here at their full size for clarity.

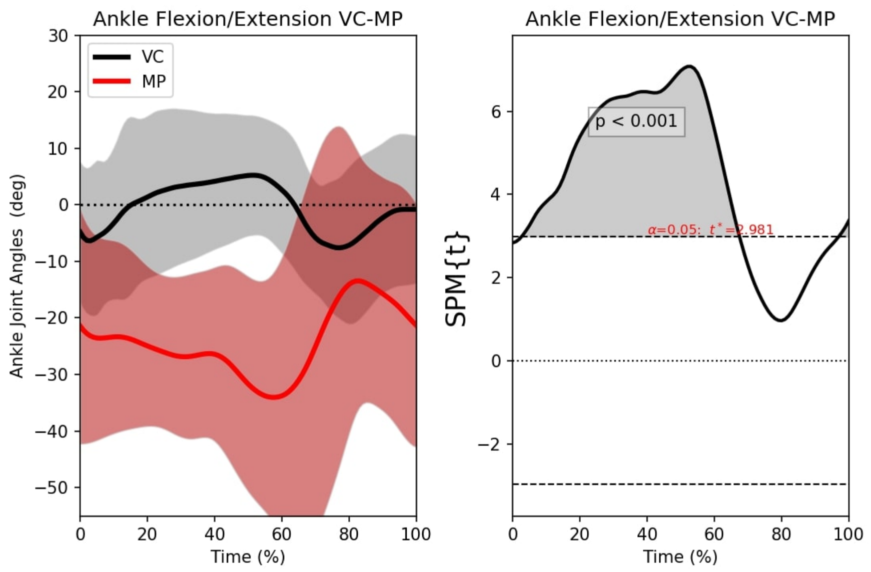

Figure S10. Statistical Parametric Mapping of the Ankle Joint Between VC and MP

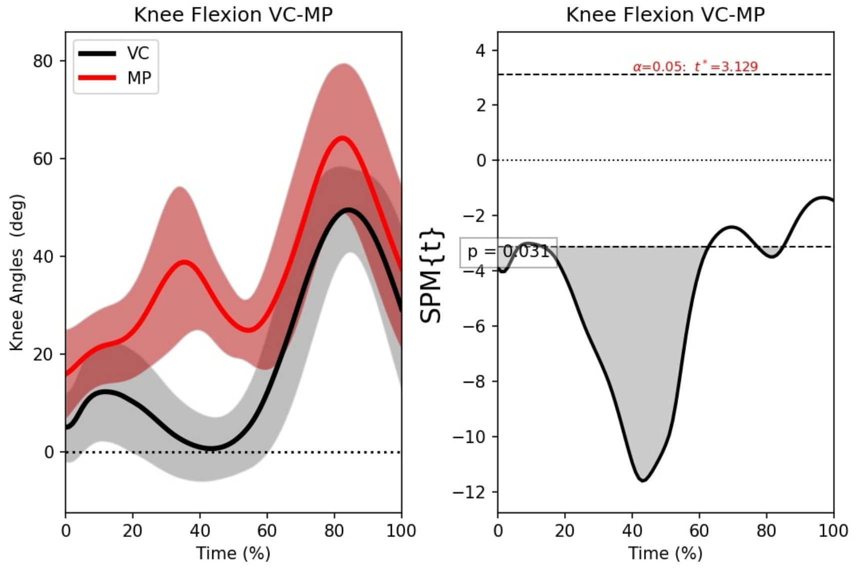

Figure S11. Statistical Parametric Mapping of the Knee Joint Between VC and MP

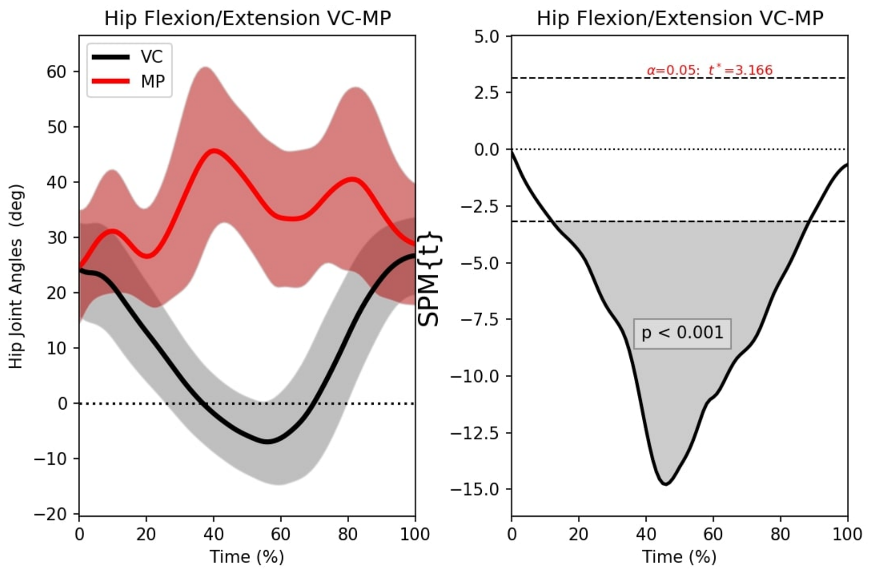

Figure S12. Statistical Parametric Mapping of the Hip Joint Between VC and MP

1.4 Openpose VS Kinovea SPM

The statistical parametric mapping between OP and KV is given here. The figures are presented here at their full size for clarity.

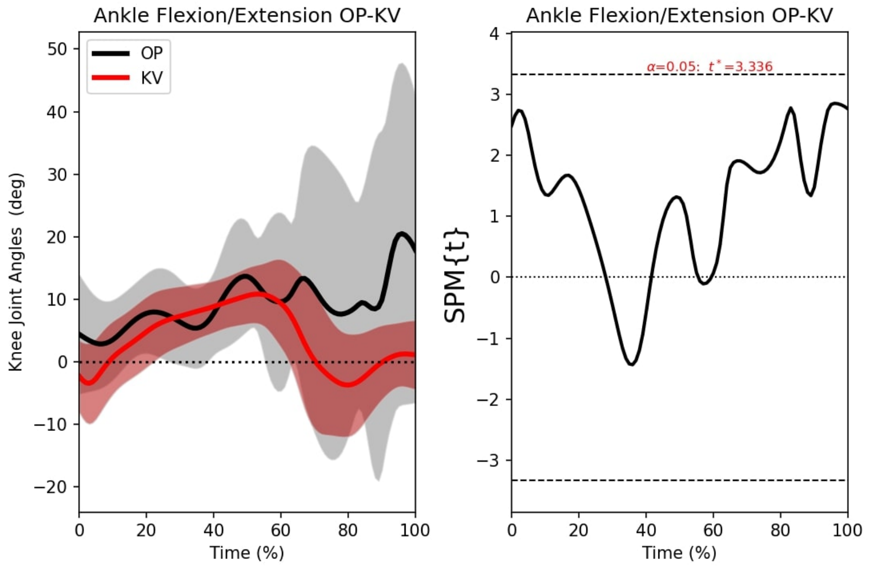

Figure S13. Statistical Parametric Mapping of the Ankle Joint Between OP and KV

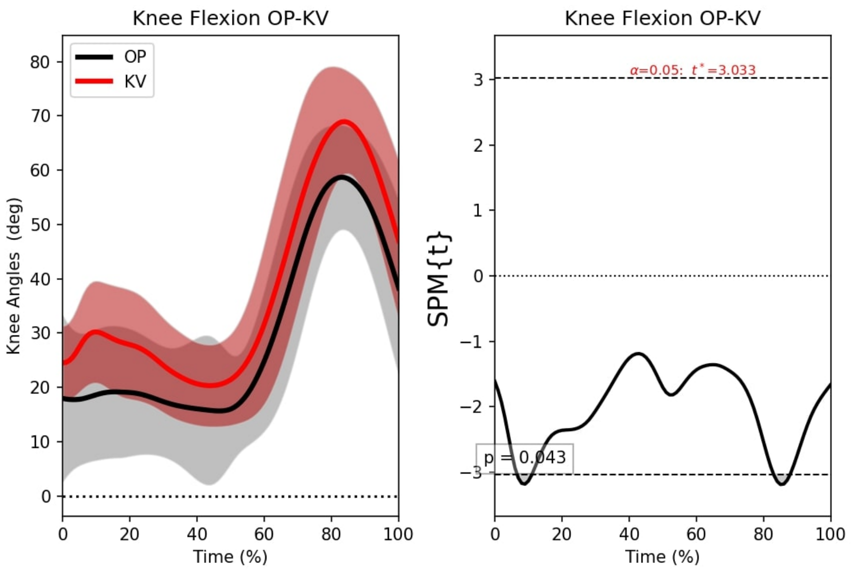

Figure S14. Statistical Parametric Mapping of the Knee Joint Between OP and KV

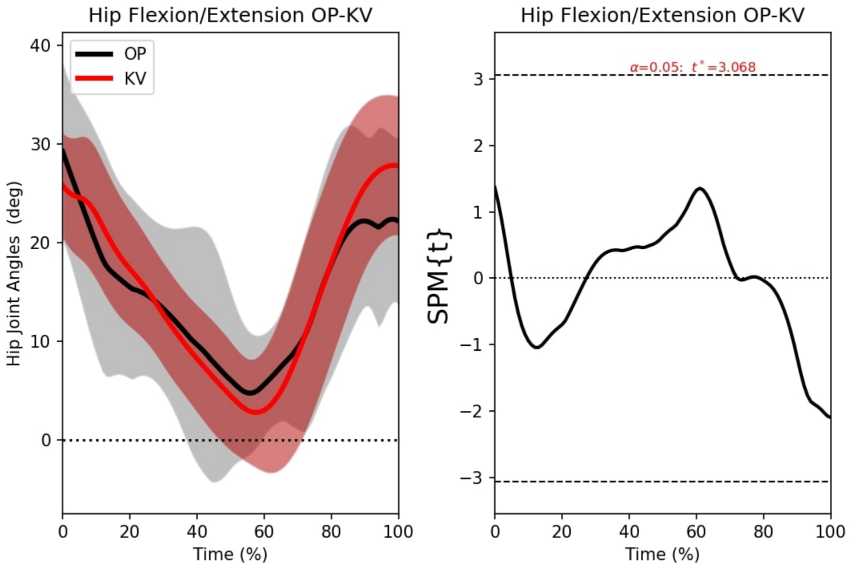

Figure S15. Statistical Parametric Mapping of the Hip Joint Between OP and KV

2 BLAND-ALTMAN GRAPHS

The B-A scatter plots are shown here between all cases. The figures are presented here at their full size for clarity.

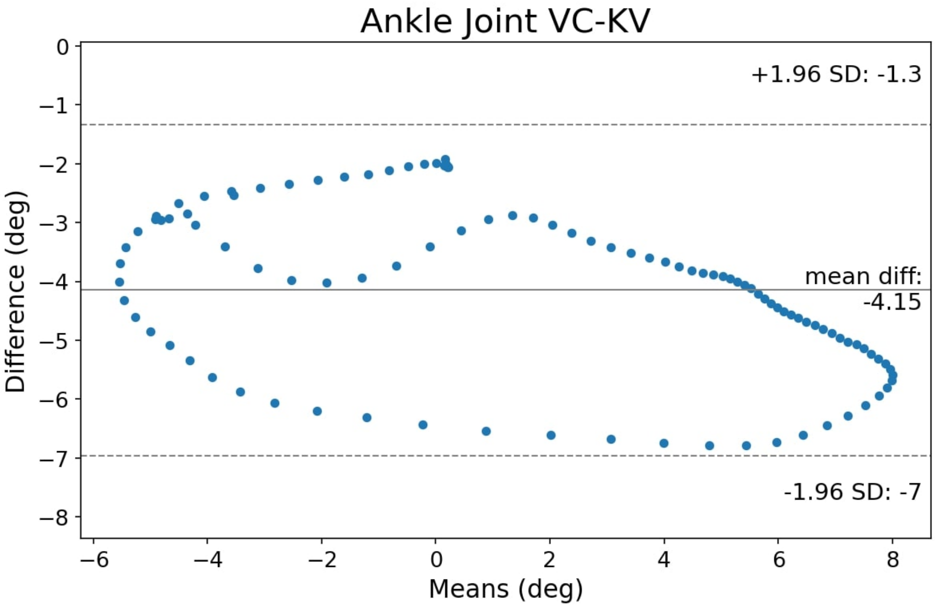

Figure S16. Band-Altman of the Ankle Joint Between VC and KV

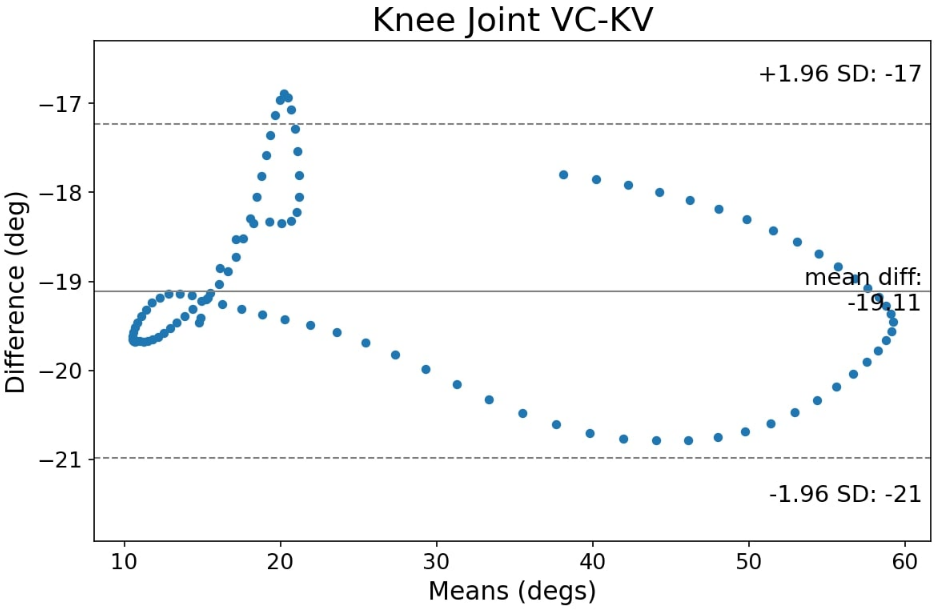

Figure S17. Band-Altman of the Knee Joint Between VC and KV

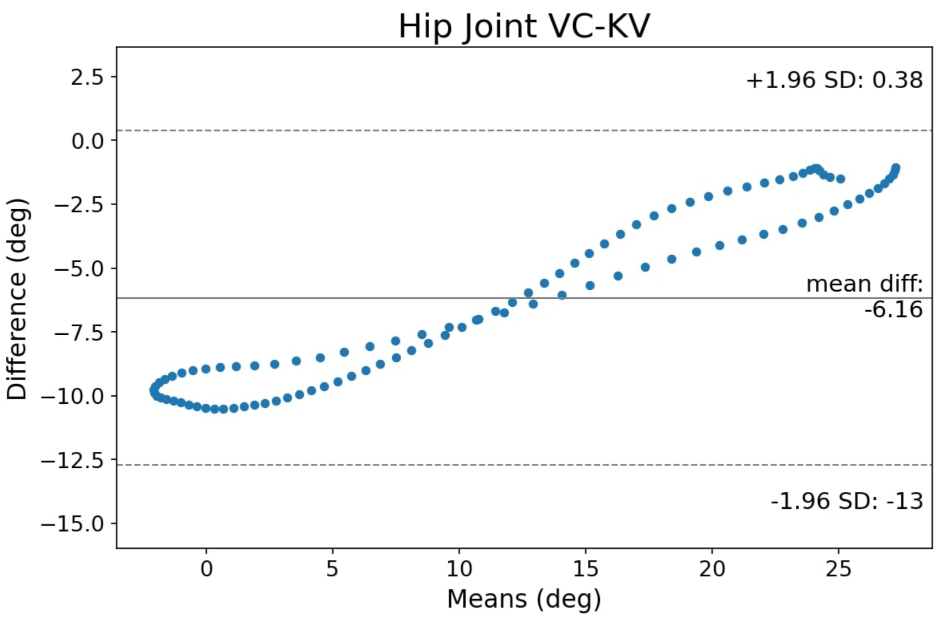

Figure S18. Band-Altman of the Hip Joint Between VC and KV

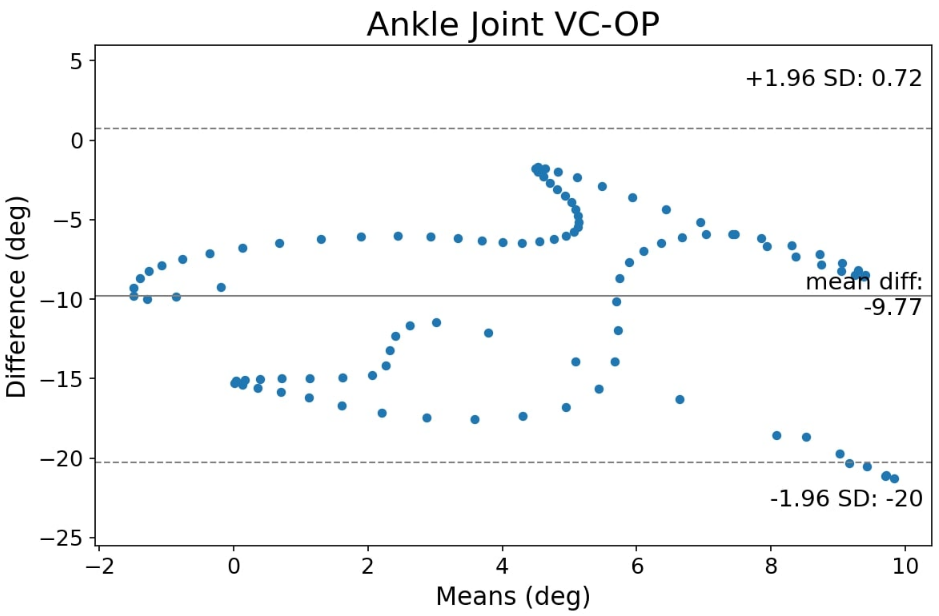

Figure S19. Band-Altman of the Ankle Joint Between VC and OP

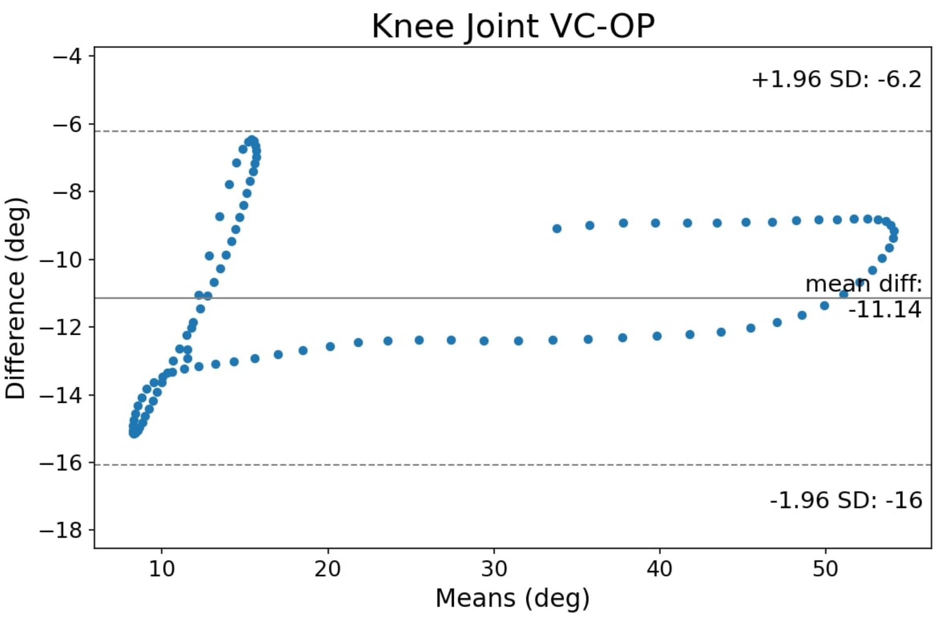

Figure S20. Band-Altman of the Knee Joint Between VC and OP

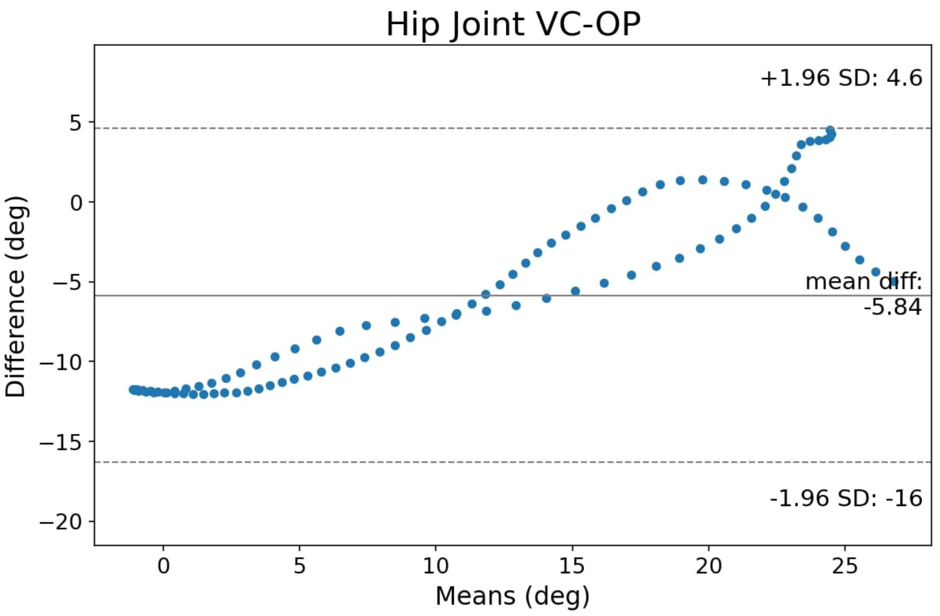

Figure S21. Band-Altman of the Hip Joint Between VC and OP

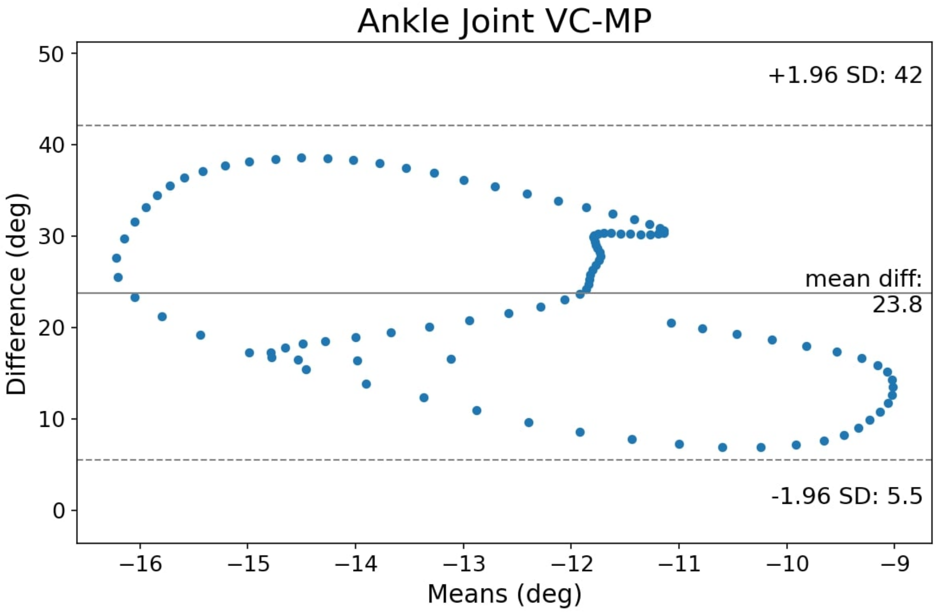

Figure S22. Band-Altman of the Ankle Joint Between VC and MP

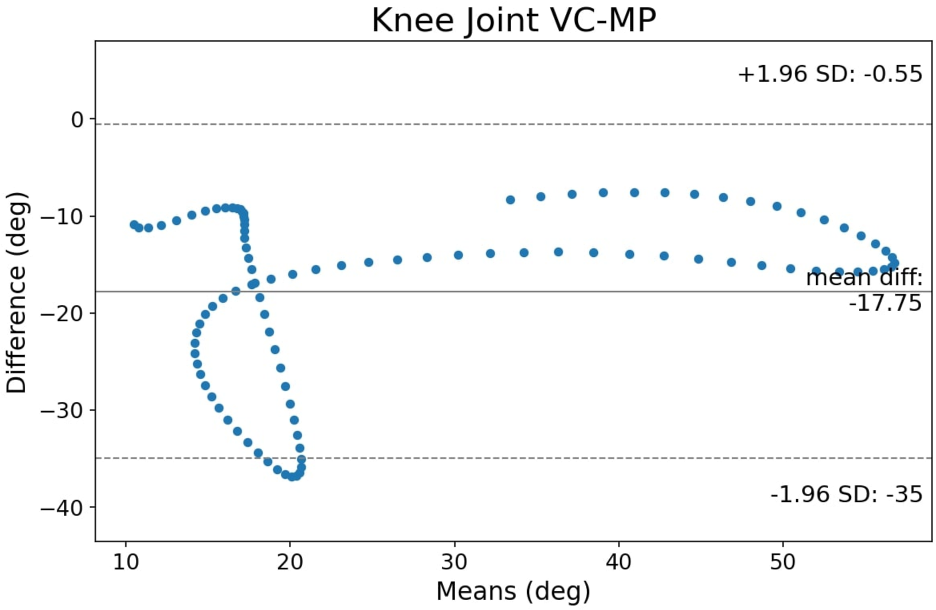

Figure S23. Band-Altman of the Knee Joint Between VC and MP

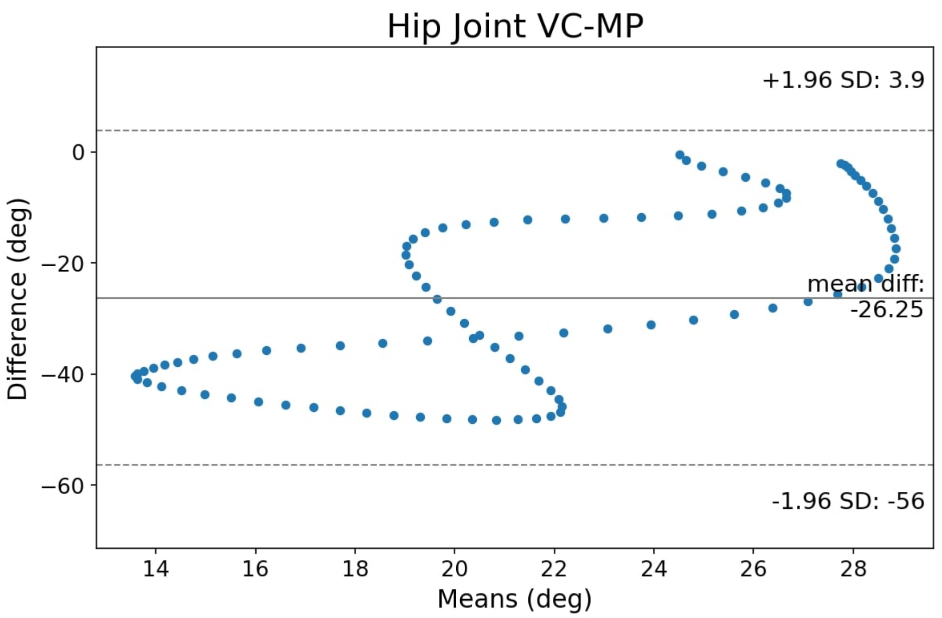

Figure S24. Band-Altman of the Hip Joint Between VC and MP

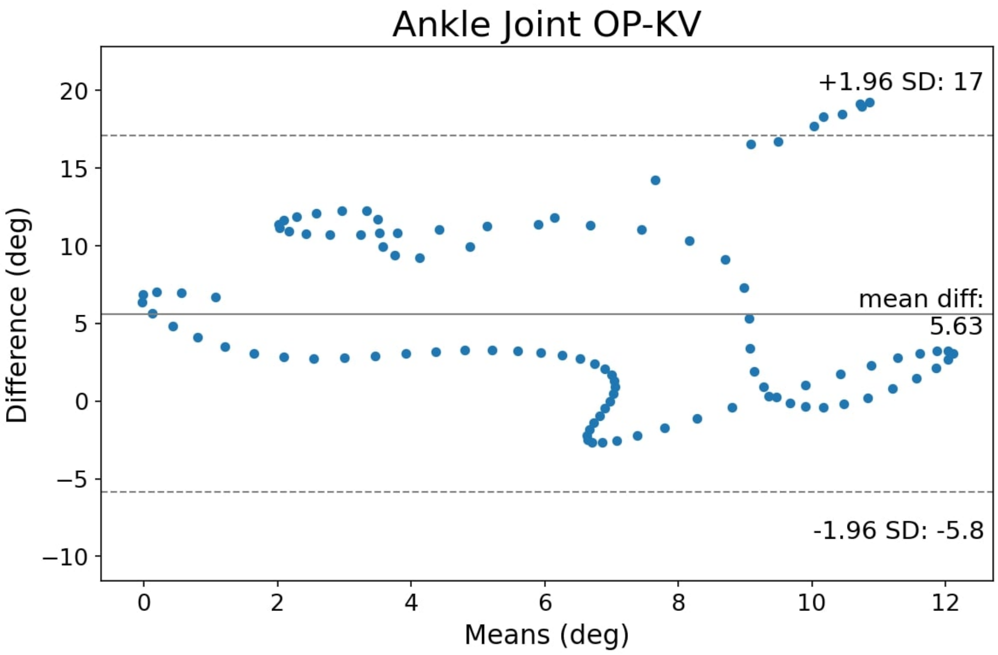

Figure S25. Band-Altman of the Ankle Joint Between VC and MP

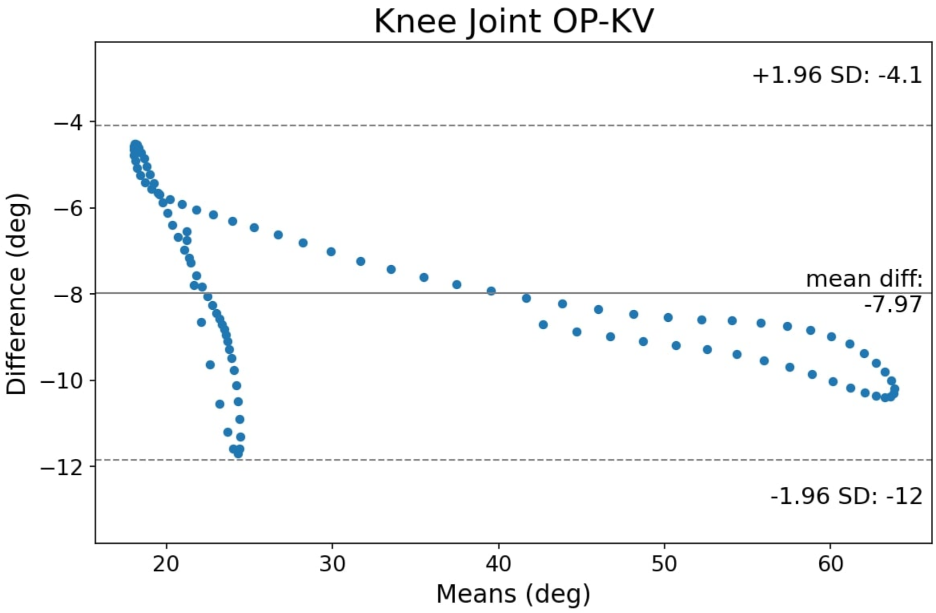

Figure S26. Band-Altman of the Knee Joint Between VC and MP

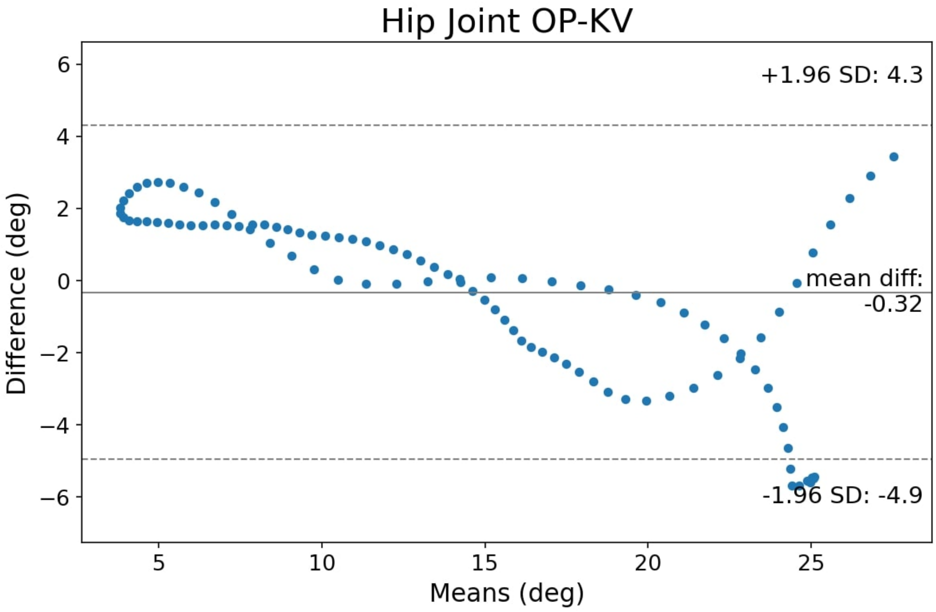

Figure S27. Band-Altman of the Hip Joint Between VC and MP
